# Supplementary material for: The Fanconi Anemia Pathway Maintains Genome Stability by Coordinating Replication and Transcription
Source: Mol Cell. 2015 Nov 5;60(3):351–61. doi: 10.1016/j.molcel.2015.09.012 (PMC4644232; doi:10.1016/j.molcel.2015.09.012)
Supplement: Document S1. Supplemental Experimental Procedures, Figures S1–S6, and Tables S1 and S2 [file mmc1.pdf]

Molecular Cell, Volume *60*

Supplemental Information

The Fanconi Anemia Pathway Maintains Genome Stability by Coordinating Replication and Transcription

Rebekka A. Schwab, Jadwiga Nieminuszczy, Fenil Shah, Jamie Langton, David Lopez Martinez, Chih-Chao Liang, Martin A. Cohn, Richard J. Gibbons, Andrew J. Deans, and Wojciech Niedzwiedz

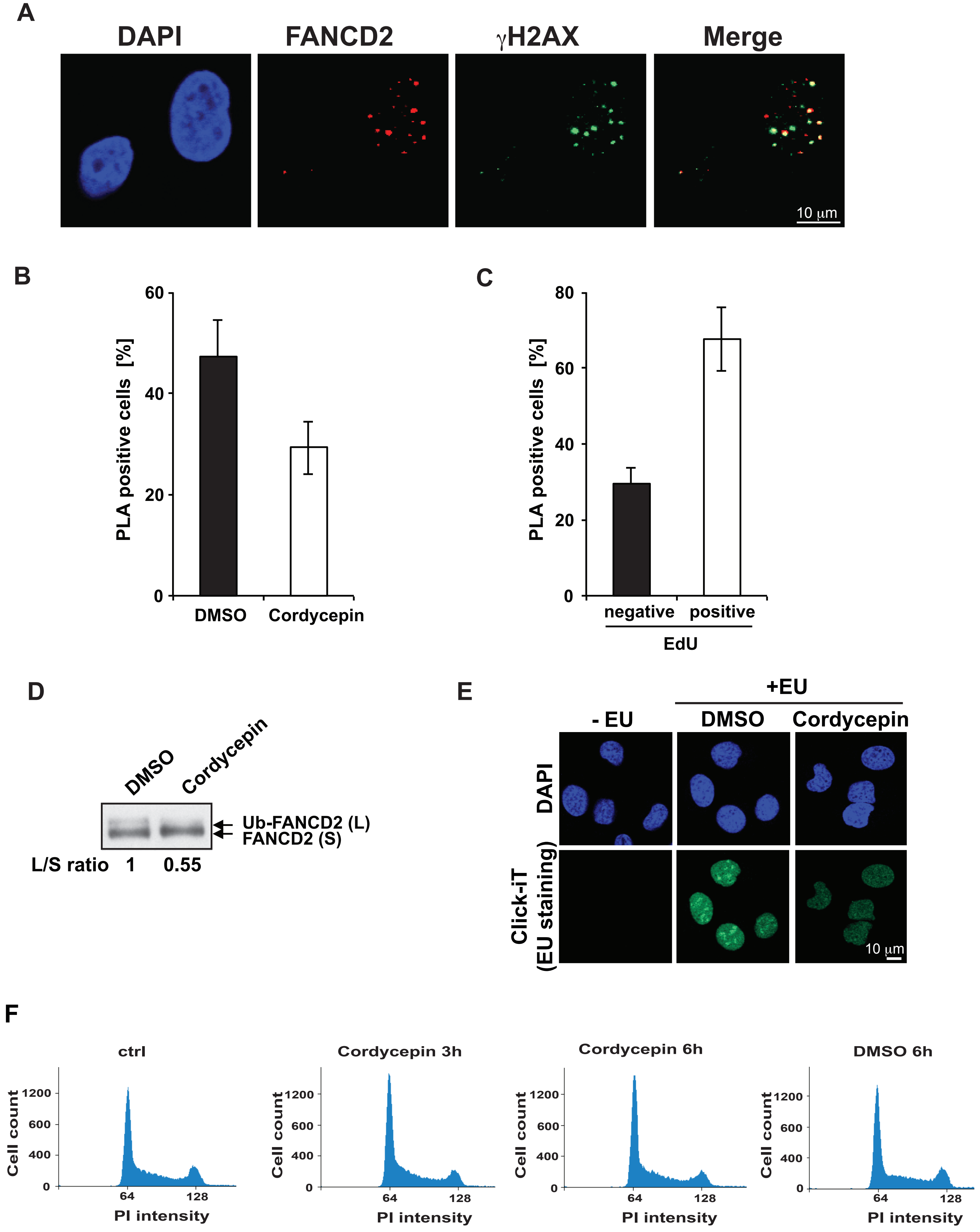

G

	G1	S	G2/M
ctrl	42.7	38.2	17.7
Cordycepin 3h	46.9	38.7	14.2
Cordycepin 6h	44.6	39.2	16.3
DMSO 3h	41.0	38.6	20.3
DMSO 6h	37.8	38.9	23.2

Figure S1

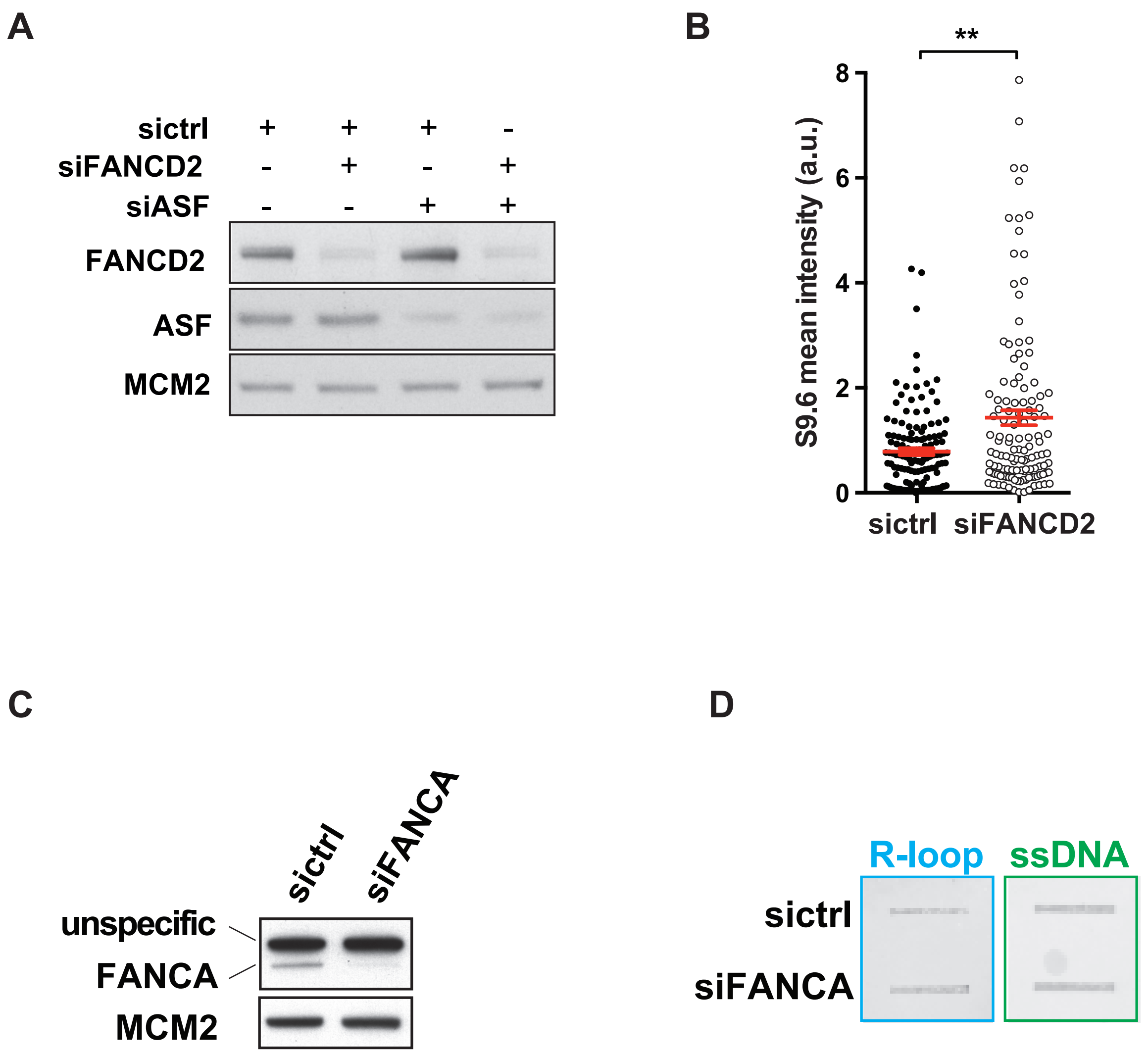
A
sictrl + + + -
siFANCD2 - + - +
siASF - - + +
FANCD2
ASF
MCM2
B
**
S9.6 mean intensity (a.u.)
0
2
4
6
8
sictrl
siFANCD2
C
sictrl
siFANCA
unspecific
FANCA
MCM2
D
R-loop
ssDNA
sictrl
siFANCA

Figure S2

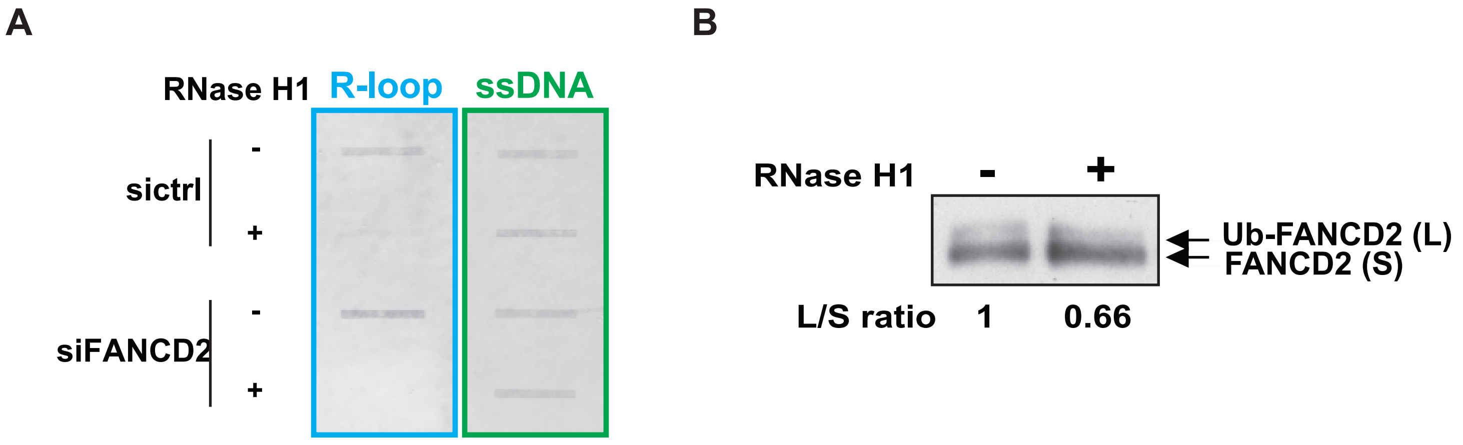

C

	G1	S	G2/M
ctrl	33.15	43.27	24.40
GFP	32.45	43.26	22.03
0.5 μg GFP-RNAse H1	32.16	41.90	25.77
1.5 μg GFP-RNase H1	32.05	43.34	24.49

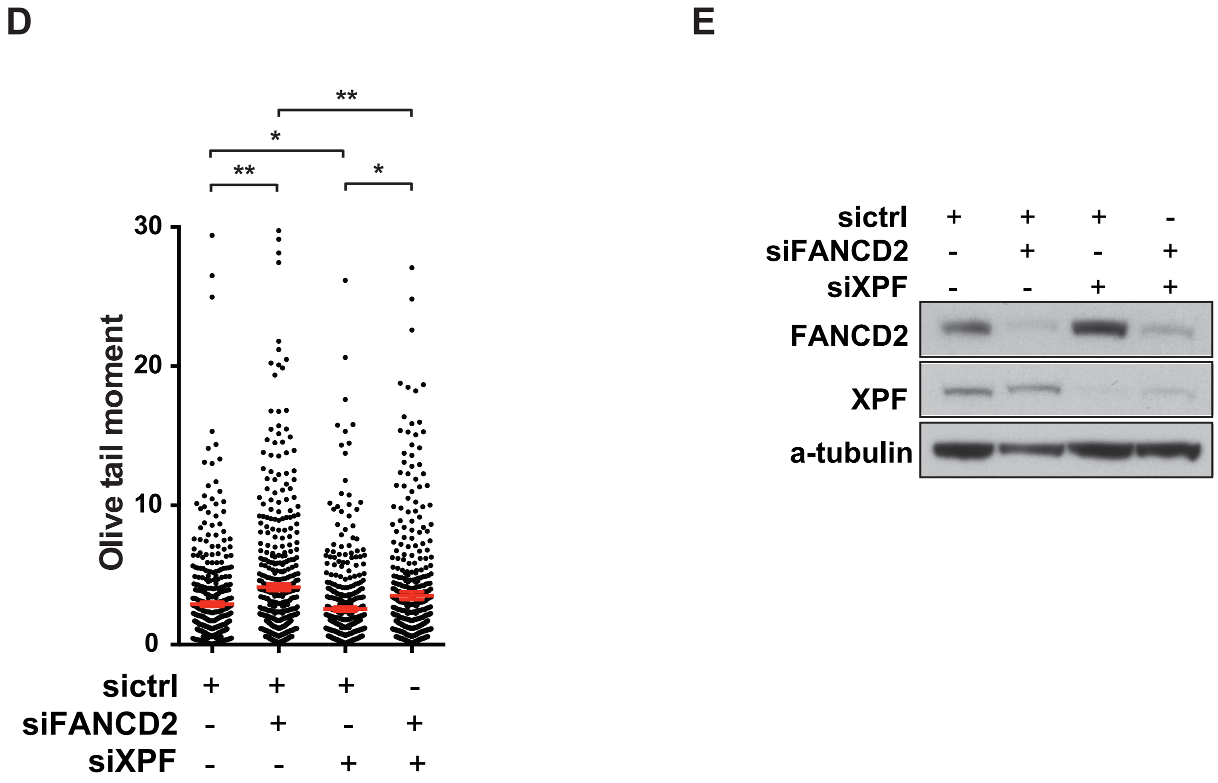

Figure S3

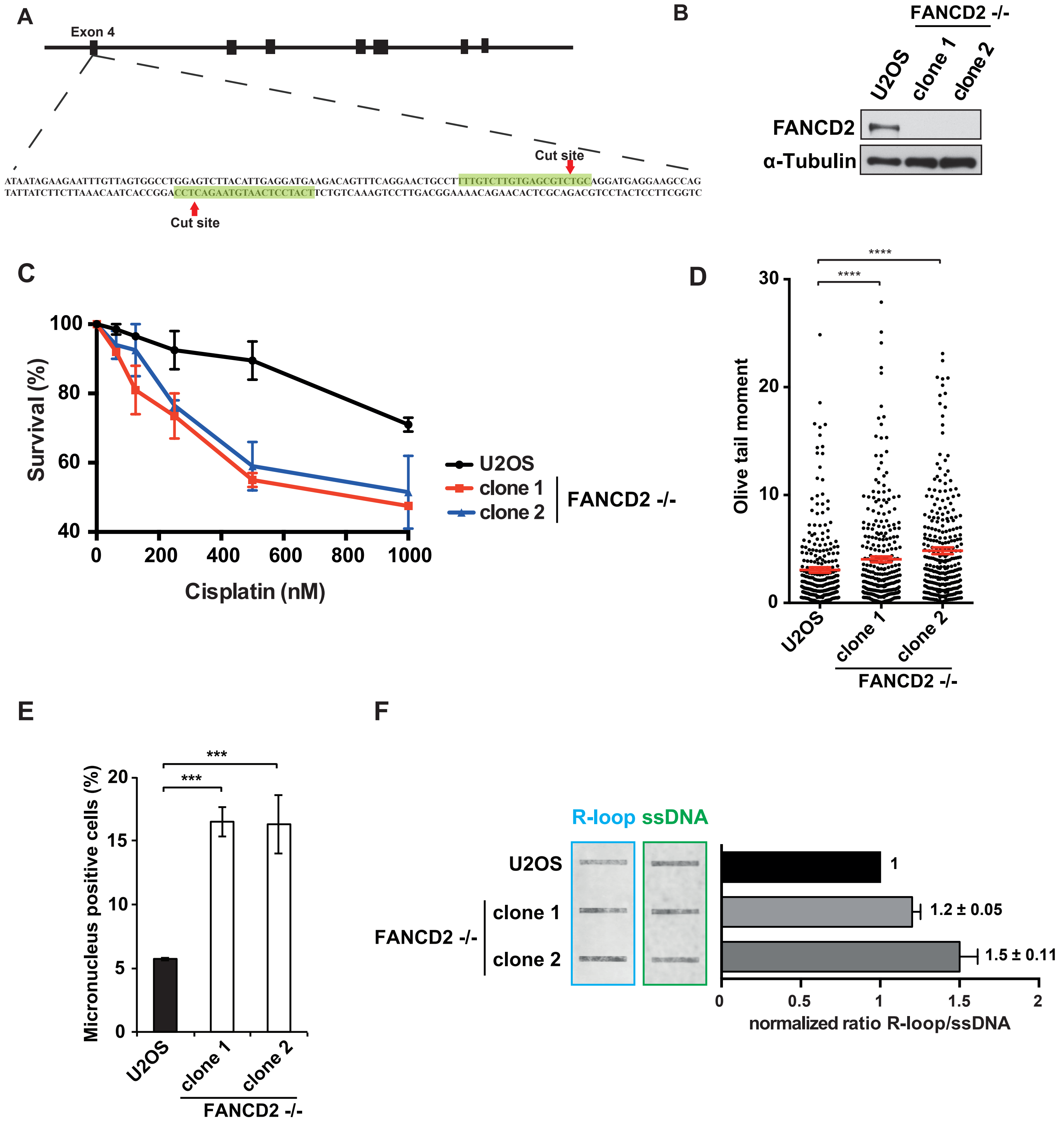

A
Exon 4
Cut site
ATAATAGAAGAATTTGTTAGTGGCCTGGAGTCTTACATTGAGGATGAAGACAGTTTCAGGAACTGCCTTTTGTCTTGTGAGCGTCTGCAGGATGAGGAAGCCAG
TATTATCTTCTTAAACAATCACCGGACCTCAGAATGTAACTCCTACTTCTGTCAAAGTCCTTGACGGAAAACAGAACACTCGCAGACGTCCTACTCCTTCGGTC
Cut site
B
FANCD2 -/-
U2OS
clone 1
clone 2
FANCD2
α-Tubulin
C
Survival (%)
100
80
60
40
0
200
400
600
800
1000
Cisplatin (nM)
U2OS
clone 1
clone 2
FANCD2 -/-
D
Olive tail moment
30
20
10
0
****
****
U2OS
clone 1
clone 2
FANCD2 -/-
E
Micronucleus positive cells (%)
20
15
10
5
0
***
***
U2OS
clone 1
clone 2
FANCD2 -/-
F
R-loop
ssDNA
U2OS
FANCD2 -/-
clone 1
clone 2
1
1.2 ± 0.05
1.5 ± 0.11
0
0.5
1
1.5
2
normalized ratio R-loop/ssDNA

Figure S4

A

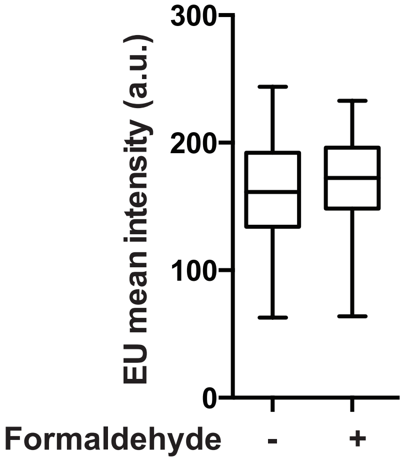

B

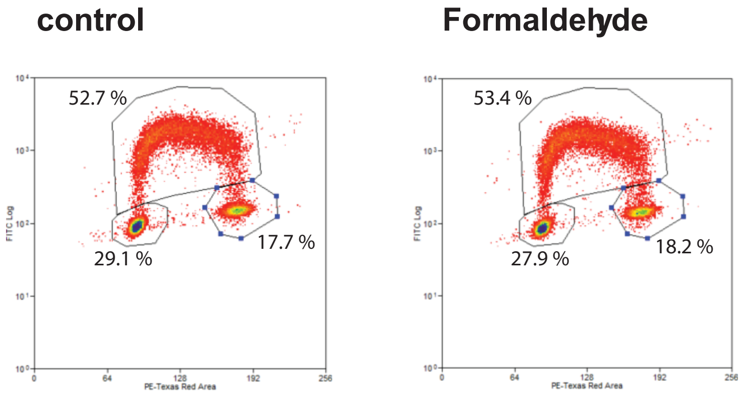

C

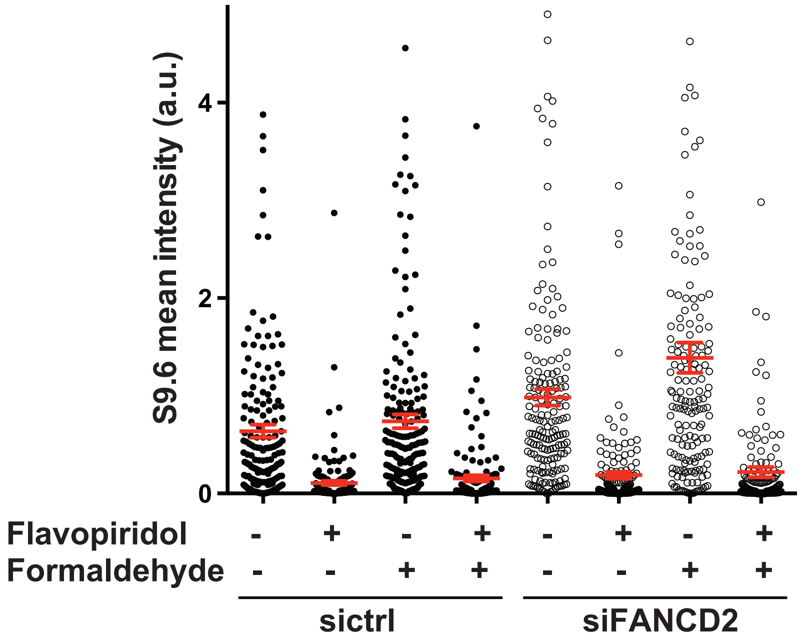

Figure S5

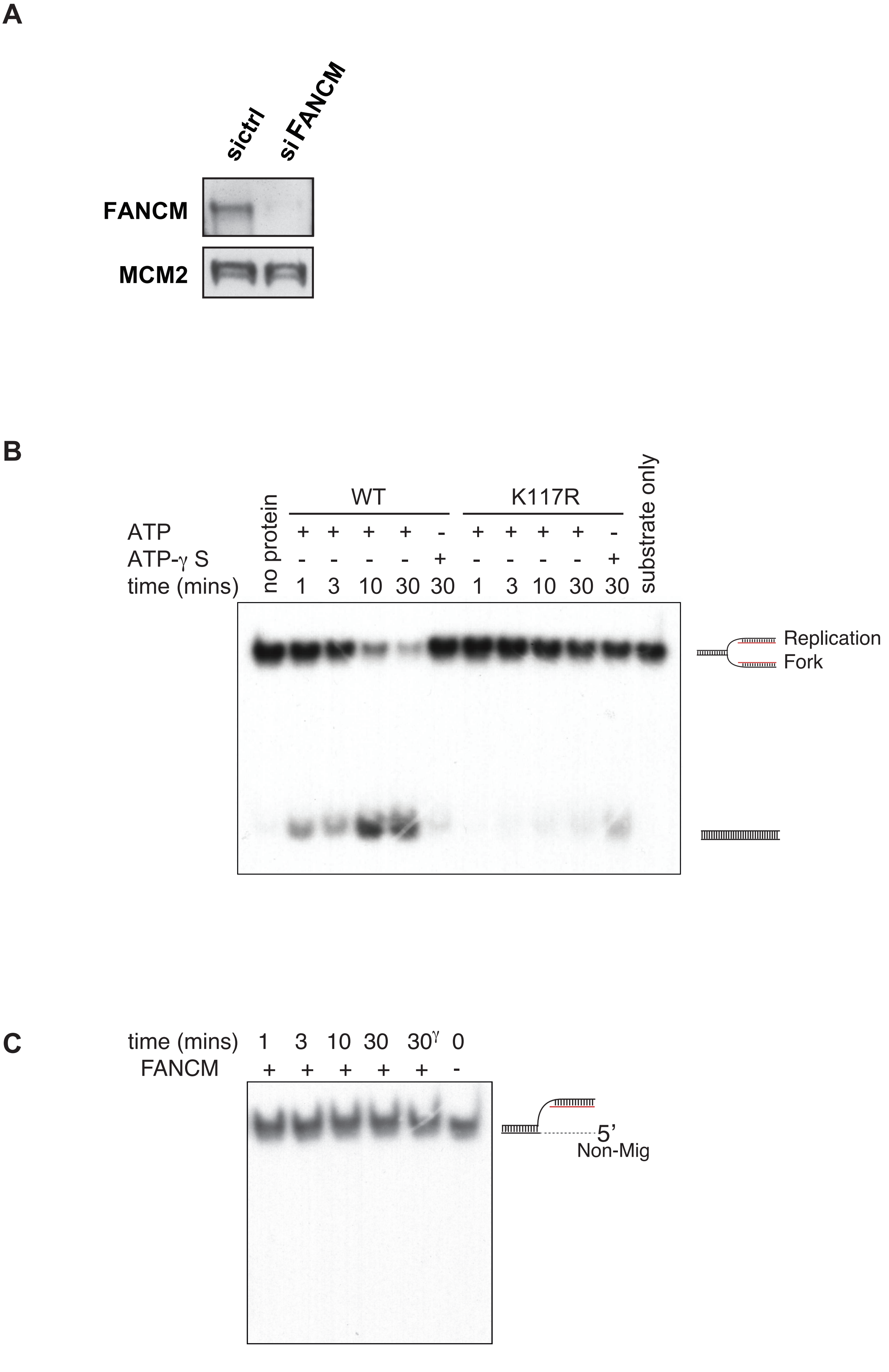
A
sictrl
si FANCM
FANCM
MCM2
B
no protein
WT
K117R
substrate only
ATP
ATP-γ S
time (mins)
1
3
10
30
30
Replication
Fork
C
time (mins)
1
3
10
30
30γ
0
FANCM
5'
Non-Mig

Figure S6

Supplementary Figures:

Figure S1, related to Figure 1 and 2. (A) Colocalization of γH2AX and FANCD2 foci in undamaged cells. Exponentially growing U2OS cells were examined by immunofluorescence using antibodies against FANCD2 and γH2AX. (B) Quantification of PLA assay using antibodies against endogenous FANCD2 and RNA polII-pS2. Graph showing frequency of PLA positive cells in the presence of DMSO or cordycepin (means and SEM, n=3). (C) Chart displaying percentage of PLA positive cells with or without EdU staining (means and SEM, n=3). (D) Effect of cordycepin treatment on FANCD2 ubiquitination. Monoubiquitination of FANCD2 was analyzed in HeLa cells treated with DMSO or 50 μM cordycepin for 2h using an antibody against the full-length protein. Densitometric analysis was used to determine the ratio between the monoubiquitinated (L) and the short form of FANCD2 (S). (E) Efficacy of cordycepin treatment. Analysis of global RNA transcription in cells treated with EU (5-ethynyl uridine) and DMSO or 50 μM cordycepin. Newly synthesized RNA is visualized using the click chemistry and Alexa Fluor 488 azide. (F) Cell cycle profiles of exponentially growing U2OS cells exposed to 50 μM cordycepin for the times indicated analyzed using flow cytometry and propidium iodide staining. (G) Percentage of cells in the indicated phases of the cell cycle.

Figure S2, related to Figure 3 and 4. (A) Western blot of whole cell lysates from U2OS cells treated with sictrl, siFANCD2 or siASF according to the legend. MCM2 serves as loading control. (B) Graph displays quantification of nuclear S9.6 staining intensities excluding nucleolar signals of the same pictures used for the analysis in Figure 4B with mean and SEM in red (two-tailed Mann-Whitney test, n=3). (C) Western blot of whole cell lysates from U2OS cells treated with control or FANCA siRNA. MCM2 is the

loading control. (B) Slot blot of genomic DNA from sictrl and siFANCA treated cells probed with the S9.6 antibody. After denaturation the DNA loading was assessed with an antibody recognizing ssDNA.

Figure S3, related to Figure 4 and 5. (A) Genomic DNA of control and FANCD2-depleted cells was treated with RNase H1 *in vitro* before blotting onto a membrane. Binding of the S9.6 antibody was assessed by IR fluorescence measurement and detection of ssDNA after denaturation serves as loading control. (B) RNase H1 decreases FANCD2 ubiquitination. Monoubiquitination of FANCD2 was analyzed in exponentially growing HeLa cells mock transfected or transfected with a cDNA coding for RNase H1 fused to GFP. An antibody against full-length FANCD2 was used to determine the ratio between monoubiquitinated FANCD2 (L) and the unmodified, short form (S). (C) Percentage of cells in the indicated phases of the cell cycle for control U2OS, U2OS cells transfected with a construct encoding GFP only, and U2OS cells transiently transfected with the indicated concentrations of plasmid DNA encoding GFP-RNase H1. (D) Comet assays of U2OS cells depleted for XPF and FANCD2 by siRNA. Individual data points of olive tail moment are plotted showing mean and SEM in red, n=3, two-tailed Mann-Whitney test. (E) Western blot confirming knock down of FANCD2 and XPF.

Figure S4, related to Figure 1, 2 and 4. (A) FANCD2 knockout generation strategy using the CRISPR-Cas9 nickase. Schematic representation of the human FANCD2 genomic locus with guide RNA sequences highlighted in green and predicted cut sites marked by red arrows. (B) Western blot of whole cell lysates from U2OS cells blotted with an anti-FANCD2 antibody. Tubulin is the loading control. (C) Survival assay of control U2OS and $FANCD2^{-/-}$ clones treated with the indicated doses of cisplatin. Survival data

represent mean +/- SEM, (n=3). (D) Comet assays of WT U2OS and FANCD2$^{-/-}$ clones. Individual olive tail moment values are plotted showing mean and SEM in red, n=3, two-tailed Mann-Whitney test. (E) Means and SEM of micronuclei positive WT U2OS and FANCD2$^{-/-}$ clones are plotted (n=3, unpaired, two-tailed Student's t-test). (F) Slot blot of genomic DNA from sictrl and FANCD2$^{-/-}$ cells probed with the S9.6 antibody. After denaturation the DNA loading was assessed with an antibody recognizing ssDNA.

Figure S5, related to Figure 5. (A) Global RNA transcription was measured in cells treated with EU in the presence and absence of formaldehyde. Fluorescence intensity measurements were derived from newly synthesized RNA visualized using the click chemistry and Alexa Fluor 488 azide. (B) Cell cycle analysis of BrdU labelled cells treated or untreated with formaldehyde. (C) Distribution of mean fluorescence intensity of individual nuclei from control and FANCD2 depleted cell in the presence or absence of formaldehyde and the transcription inhibitor flavopiridol (mean and SEM in red, two-tailed Mann-Whitney test, n=3).

Figure S6, related to Figure 6. (A) Western blot of control and siFANCM treated U2OS cell lysates probed with an antibody against FANCM; MCM2 was used to assess equal protein loading. (B) FANCM and FAAP24 unwind a migratable replication fork structure (control). Unwinding is inhibited in the presence of FANCM K117R helicase-dead mutant protein or non-hydrolysable ATP (ATP-γ-S). (C) FANCM and it's binding partner FAAP24 do not resolve a non-migratable DNA:RNA flap structure.

Supplementary experimental procedures

Antibodies. Anti-MCM2 (ab4461, Abcam), anti-MCM2-pS108 (AHP1525, AbD Serotec), anti-RPA2 (Ab-2; NA18, Calbiochem), anti-ASF (324500, Invitrogen), anti-RPA2-pS4/8 (A300-245A, Bethyl), anti-RPA2-pS33 (A300-246A, Bethyl), anti-PCNA (PC10; sc-56, Santa Cruz), anti-H3 (ab1791, Abcam), anti-γ-H2AX (JBW301; 05-636, Millipore), anti-FANCD2 for western blotting (FI17; sc-20022, Santa Cruz), anti-FANCD2 for IF was a gift from K.J. Patel, anti-FANCA (6512, FARF), anti-FANCM (Cancer Research Technology), anti-GFP (11814460001, Roche), anti-RNA polymerase II (05-623, Millipore), anti-RNA polymerase II H5 (MMS-129R, Covance), anti-single stranded DNA (MAB3034, Millipore), α-tubulin (T5168, Sigma-Aldrich), anti-p53 (phospho-S15, Abcam), anti-p21 (Santa Cruz Biotech), XPF (ab17798, Abcam). The anti-DNA:RNA hybrid (S9.6) antibody was from S9.6 hybridoma cells grown in Iscove's modified Dulbecco's medium with 1x HT solution (HAT minus aminopterine) and 20% horse serum until they reached the stationary phase and began to die. The medium was then filtered and sodium azide added. The S9.6 containing medium was used with no further purification.

Transcription assay. Inhibition of transcription after cordycepin treatment was assessed with the Click-it RNA Imaging Kit and Alexa Fluor 488 azide (Invitrogen). U2OS cells were grown on cover slips and following treatment with 50 μM cordycepin (Sigma-Aldrich) for 2 h, cells were incubated with 1 mM EU for 1 h and then fixed with 4% paraformaldehyde for 10 min. Click reactions were performed in accordance with the manufacturer's instructions.

Western blot analysis. Whole cell protein extracts were prepared by lysing washed cells in denaturing buffer (9 M urea, 150 mM 2-mercaptoethanol, 50 mM Tris-HCl pH 7.3) and subsequently sonicated to shear genomic DNA. FANCM was separated on 7% Tris-glycine mini gels and FANCD2 ubiquitination was assessed on 3-8% NuPAGE Tris-Acetate mini gels (Life Technologies). For all other proteins 4-12% NuPAGE Bis-Tris mini gels (Life Technologies) were used. Gels were transferred onto nitrocellulose membranes (GE Healthcare). HRP conjugated secondary antibodies (Dako) and Immobilon enhanced chemiluminescence reagents (Millipore) were used to visualize

antigens. Intensities of the bands of the long and short forms of FANCD2 were measured using ImageJ.

Proximity ligation assay (PLA). U2OS cells were grown on cover slips, washed with PBS and fixed with 4% paraformaldehyde for 10 min. After permeabilization with 0.5% Triton X-100 for 5 min, cells were blocked with 10 % FBS for 1 h and then incubated with anti-FANCD2 (1:750) alone as negative control or in combination with either anti-RNA polymerase (1:600) or anti-RNA polymerase II H5 (phospho-S2) (1:600) in 0.1% FBS for 2 h. Binding of PLA probes, ligation and amplification was performed with reagents from the Duolink In Situ Kit (Sigma-Aldrich) according to manufacturer's instructions.

S-phase analysis of PLA positive cells. Cells were labelled with 10 μM EdU for 30 min and then fixed with 10% PFA for 10 min. S-phase cells were visualized using the Click-iT EdU imaging kit from Molecular Probes according to manufacturers instructions. Subsequently, PLA assays were performed as described.

Cell cycle analysis. U2OS cells were treated with DMSO or 50 μM cordycepin for 3 and 6 h, trypsinized and washed with PBS containing 2% FBS. Cells were resuspended in 0.5 ml PBS with 2% FBS and fixed with ice cold 70% ethanol. Cells were then washed with PBS, incubated with 2 M HCl for 30 min, washed twice with PBS containing 0.5% Tween-20 and 1% BSA, and subsequently stained with propidium iodide solution (PBS, 0.5% Tween-20, 1% BSA, 20 mg/ml propidium iodide, 250 mg/ml RNase A) for 30 min at 37°C. Cell cycle profiles were analyzed using CyAn ADP Analyzer (Beckman Coulter) and Summit 4.3 software.

Cell proliferation assay. RNAi treated U2OS cells were plated in 96 well plates and 4 h before fluorometric measurements, alamarBlue (Bio-Rad) was added to the samples according to manufacturer's instructions.

Alkaline comet assay. U2OS cells were trypsinized, washed and resuspended in PBS at a density of 0.5 x 10^6 cells/ml. Cell suspensions were mixed at a 1:1 ratio with 1.5 % low melting point agarose Type VII (Sigma-Aldrich) and cast on a microscope slide precoated with 0.5% agarose Type IA (Sigma-Aldrich). After solidification, slides were

incubated in lysis buffer (2.5 M NaCl, 100 mM EDTA, 10 mM Tris, pH 10, 1% Triton X-100) for 2 h at 4°C. After lysis, slides were placed in a horizontal electrophoresis tank covered by electrophoresis buffer (1 mM EDTA, 300 mM NaOH) and incubated for 40 min. Electrophoresis was performed at 1V/cm distance between electrodes for 40 min. Slides were washed twice with 0.4 M Tris-HCl pH 7.5 and twice with distilled water before immersing in 70% ethanol for 5 min. Slides were then air-dried and subsequently stained with SybrGold (Invitrogen). Comets were analyzed with Komet 6 (Andor) software.

Supplemental Table 1, related to experimental procedures: Oligos used in the generation of DNA:RNA or DNA:DNA hybrid structures. Complementary sequences are colour coded:

XOmig1	5'ACGCTGCCGAATTCTACCAGTGCCTTGCTAGGACATCTTTGCCCACCTGCAGGTTCACCC3'
XOmig 2	5'GGGTGAACCTGCAGGTGGGCAAAGATGTCCCAGCAAGGCACTGGTAGAATTCGGCAGCGT3'
XOFlap3 R	RNA-5'GGACAUCUUUGCCCACCUGCAGGUUCACCC3'
XOFlap5 R	RNA-5'GGGUGAACCUGCAGGUGGGCAAAGAUGUCC3'
XO2non	5'CGATAGTCGGATCCTCTAGACAGCTCCATGTAGCAAGGCACTGGTAGAATTCGGCAGCGT3'

Supplementary Table 2, related to experimental procedures: 2 step annealing procedure for generation of different DNA:DNA and DNA:RNA hybrid structures

Substrate	Step 1		Step 2	
	Oligos	Temp	Oligos	Temp
Migratable Replication fork	(A) XOmig1 + XOFlap5R, (B) XOmig2 + XOFlap3R	100C, O/N	Mix (A) and (B)	37C, 1hr
Migratable 5' Flap	(A) XOmig1 + XOFlap5R,	100C, O/N	Mix (A) and XOmig2	37C, 1hr
Migratable 3' Flap	(B) XOmig2 + XOFlap3R	100C, O/N	Mix (B) and XOmig2	37C, 1hr
dsDNA	(C) XOmig1 + XOmig2	100C, O/N	-	-
Non migratable 5'Flap	(D) XOmig1 + XO1non + XOFlap5R	100C, O/N	-	-
