# Supplementary material for: The Fanconi Anemia Pathway Maintains Genome Stability by Coordinating Replication and Transcription
Source: Mol Cell. 2015 Nov 5;60(3):351–61. doi: 10.1016/j.molcel.2015.09.012 (PMC4644232; doi:10.1016/j.molcel.2015.09.012)
Supplement: Document S2. Article plus Supplemental Information [file mmc2.pdf]

Molecular Cell

Article

The Fanconi Anemia Pathway Maintains Genome Stability by Coordinating Replication and Transcription

Graphical Abstract

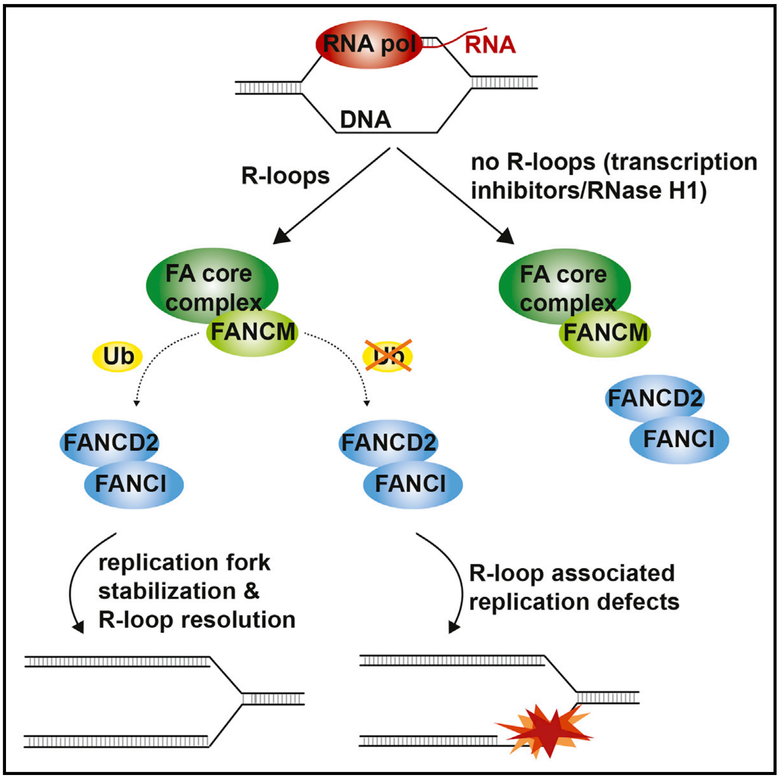

Highlights

- Replication and transcription collisions cause genome instability in FA
- A functional FA pathway protects cells from unscheduled accumulation of R-loops
- Transcription inhibition or R-loop removal restores normal replication in FA cells
- FANCM resolves R-loops via its translocase activity

Authors

Rebekka A. Schwab, Jadwiga Nieminuszczy, Fenil Shah, ..., Richard J. Gibbons, Andrew J. Deans, Wojciech Niedzwiedz

Correspondence

wojciech.niedzwiedz@imm.ox.ac.uk

In Brief

Schwab et al. show that the FA pathway prevents DNA lesions caused by conflicts between replication and transcription and by transcription-associated DNA:RNA hybrids (R-loops). FA proteins can stabilize stalled replication forks, and FANCM resolves R-loops via its translocase activity.

Schwab et al., 2015, Molecular Cell *60*, 351–361
November 5, 2015 ©2015 Elsevier Inc.
http://dx.doi.org/10.1016/j.molcel.2015.09.012

Molecular Cell
Article

The Fanconi Anemia Pathway Maintains Genome Stability by Coordinating Replication and Transcription

Rebekka A. Schwab,[1] Jadwiga Nieminuszczy,[1] Fenil Shah,[2] Jamie Langton,[1] David Lopez Martinez,[3] Chih-Chao Liang,[3] Martin A. Cohn,[3] Richard J. Gibbons,[4] Andrew J. Deans,[2] and Wojciech Niedzwiedz[1,*]
[1]Department of Oncology, Weatherall Institute of Molecular Medicine, University of Oxford, John Radcliffe Hospital, Oxford OX3 9DS, UK
[2]Genome Stability Unit, St. Vincent's Institute, Fitzroy, VIC 3065, Australia
[3]Department of Biochemistry, University of Oxford, Oxford OX1 3QU, UK
[4]Medical Research Council Molecular Haematology Unit, Weatherall Institute of Molecular Medicine, University of Oxford, John Radcliffe Hospital, Oxford OX3 9DS, UK
*Correspondence: wojciech.niedzwiedz@imm.ox.ac.uk
http://dx.doi.org/10.1016/j.molcel.2015.09.012

SUMMARY

DNA replication stress can cause chromosomal instability and tumor progression. One key pathway that counteracts replication stress and promotes faithful DNA replication consists of the Fanconi anemia (FA) proteins. However, how these proteins limit replication stress remains largely elusive. Here we show that conflicts between replication and transcription activate the FA pathway. Inhibition of transcription or enzymatic degradation of transcription-associated R-loops (DNA:RNA hybrids) suppresses replication fork arrest and DNA damage occurring in the absence of a functional FA pathway. Furthermore, we show that simple aldehydes, known to cause leukemia in FA-deficient mice, induce DNA:RNA hybrids in FA-depleted cells. Finally, we demonstrate that the molecular mechanism by which the FA pathway limits R-loop accumulation requires FANCM translocase activity. Failure to activate a response to physiologically occurring DNA:RNA hybrids may critically contribute to the heightened cancer predisposition and bone marrow failure of individuals with mutated FA proteins.

INTRODUCTION

Replication of the human genome is a complex process requiring orchestrated activation and maintenance of replication forks emanating from thousands of origins of replication during S-phase. Replication forks stall when they encounter obstacles on the DNA, upon which they require swift processing to prevent their disassembly, resulting in DNA damage. Such collapsed replication forks can contribute to spontaneous recombination events and genomic instability, a hallmark of cancer (Aguilera and Gómez-González, 2008). Faithful DNA replication requires several factors, including proteins of the Fanconi anemia (FA) pathway. To date, 18 FA genes (FANCA-T) have been identified, and homozygous inactivation of any FA gene product leads to the pediatric syndrome Fanconi anemia, characterized by progressive bone marrow failure, spontaneous chromosomal instability, and high cancer predisposition. Functionally, the FA pathway can be divided into at least three different sub-complexes, the largest of which is the core complex consisting of the FANCA, FANCB, FANCC, FANCE, FANCF, FANCG, FANCL, and FANCM gene products. The core complex, together with the E2 ubiquitin-conjugating enzyme FANCT/UBE2T, have a critical role in activating the FA pathway through monoubiquitination of the FANCD2 and FANCI proteins. This, in turn, promotes DNA repair through the specialized downstream Fanconi proteins FANCD1/BRCA2, FANCN/PALB2, FANCJ/BRIP1, FANCO/RAD51C, FANCP/SLX4, FANCQ/XPF/ERCC4, and FANCS/BRCA1 (Hira et al., 2015; Kee and D'Andrea, 2012; Kottemann and Smogorzewska, 2013; Rickman et al., 2015; Walden and Deans, 2014; Wang, 2007). Cells from FA patients are hypersensitive to DNA interstrand crosslinking (ICL) agents, potent inhibitors of both DNA replication and transcription. Accordingly, it has been proposed that the FA pathway has a major role in responding to replication stress by facilitating the resolution of DNA lesions arising during DNA replication (Constantinou, 2012; Knipscheer et al., 2009; Kottemann and Smogorzewska, 2013). Recently, work from the Patel group (Langevin et al., 2011) has identified simple aldehydes that can arise endogenously from processes of cellular metabolism as a potent source of DNA damage that requires action of the FA proteins. Mice with combined deficiency for FANCD2 or FANCA and the aldehyde-catabolizing enzyme Aldh2 show developmental defects and early onset of acute leukemia (Langevin et al., 2011; Oberbeck et al., 2014). However, it is unclear how aldehydes confer their toxicity because mice mutually deficient for Aldh2 and the DNA translesion synthesis polymerase Rev1, which cooperates with FA proteins in the same pathway for ICL repair (Niedzwiedz et al., 2004), do not develop any of the phenotypes observed in FANCA/Aldh2-deficient mice (Oberbeck et al., 2014). Therefore, identifying the endogenous substrate that activates the FA pathway under normal growth conditions remains one of the key questions critical for the understanding of this devastating disease.

During transcription, nascent RNA can form hydrogen bonds with one strand of the DNA double helix, leading to the formation

of DNA:RNA hybrids (R-loops). R-loop formation has been described in vivo, and its physiological functions include class switch recombination, bacterial and mitochondrial replication, and protection against DNA methylation at CpG island promoters (Aguilera and García-Muse, 2012; Skourti-Stathaki and Proudfoot, 2014). Persistent R-loops could stall replication forks driving genome instability, which is fundamental to cancer and other diseases (Bhatia et al., 2014; Lecona and Fernández-Capetillo, 2014). Here we show that conflicts between replication and transcription and also transcription-associated DNA:RNA hybrids are crucial endogenous DNA lesions that require action of the FA proteins. In particular, we provide evidence that a functional FA pathway protects cells from unscheduled accumulation of such hybrids and that its loss results in an increased level of DNA damage and spontaneous chromosomal instability, both hallmarks of FA patients. Accordingly, inhibition of transcription or removal of excess DNA:RNA hybrids by expression of RNase H1 suppresses increased replication fork stalling and DNA damage occurring in FA-depleted cells. At the mechanistic level, we show that FANCM, the most highly conserved protein in the FA pathway, resolves DNA:RNA hybrids via its intrinsic translocase activity. Unexpectedly, we also found that aldehydes induce DNA:RNA hybrid formation in FANCD2-depleted cells, suggesting a mechanism by which by-products of cellular metabolism, such as simple aldehydes, could exert their toxic effect on our genome. Therefore, we propose that DNA:RNA hybrids are endogenous and physiological substrates of the FA pathway and that, by suppressing excessive DNA:RNA hybrid formation, the FA pathway ensures faithful genome duplication.

RESULTS

The FA Pathway Facilitates Accurate Replication under Normal Growth Conditions

Hypersensitivity to agents that impede the progression of replisomes is a hallmark of FA, and, consequently, we and others have found that the FA pathway plays a role in the response to replicative stress (Knipscheer et al., 2009; Lossaint et al., 2013; Schlacher et al., 2012; Schwab et al., 2010). Accordingly, upon treatment with replication inhibitors, a central component of the FA pathway, FANCD2, is activated by monoubiquitination in an ATR-dependent manner (Andreassen et al., 2004). Subsequently, FANCD2 is targeted to damaged replication forks (Lossaint et al., 2013) and forms foci colocalizing with the DNA repair proteins γH2AX, BRCA1, and RAD51 (Montes de Oca et al., 2005; Taniguchi et al., 2002). Interestingly, FANCD2 is also monoubiquitinated and forms foci that colocalize with γH2AX in unchallenged cells (Taniguchi et al., 2002; Figure S1A). This suggests that FANCD2 is also required for dealing with replicative stress that arises in cells undergoing normal cell cycle progression. To test this, we analyzed DNA replication in control and FANCD2-depleted cells under normal growth conditions using the DNA fiber technique (Blackford et al., 2012; Schwab and Niedzwiedz, 2011). To determine the impact of FANCD2 depletion on global replication fork dynamics, we measured the lengths of sister fork tracts. Sister forks emanating from the same origin of replication and traveling in opposite directions typically display similar replication rates (Conti et al., 2007). Consequently, differences in tract lengths indicate that individual forks are more prone to stalling. We noticed a significant increase in asymmetric sister forks in FANCD2-downregulated cells compared with control cells (Figure 1A), suggesting a widespread perturbation of the normal replication program. In support of this notion, we also found increased phosphorylation of MCM2 on Ser-108 and RPA on Ser-33 (Figure 1B) in FANCD2 knockdown cells, both markers of replicative stress (Cortez et al., 2004; Sirbu et al., 2011). Failure to restart stalled forks is a strong signal for DNA damage (Schlacher et al., 2012), and, accordingly, western blot analysis showed increased γH2AX and phosphorylation of RPA2 at Ser-4/Ser-8 in FANCD2 knockdown cells (Figure 1C). These modifications are associated with DNA double-strand breaks (Sartori et al., 2007), and, therefore, we examined DNA integrity by single-cell gel electrophoresis. Cells with FANCD2 knockdown showed a significant increase in DNA breaks compared with control cells (Figure 1D). DNA damage is a precursor of genomic instability, and, in line with this, we found a greater number of cells with micronuclei in FANCD2-depleted cells (Figure 1E), a phenotype also observed in FA patient cells (Heddle et al., 1978). Moreover, *FANCC*$^{-/-}$ mouse embryonic fibroblasts (MEFs) as well as *FANCA*$^{-/-}$ mouse hematopoietic stem cells also display signs of replisome instability and activation of the DNA damage response (DDR) in unchallenged cells (Luebben et al., 2014; Walter et al., 2015). Taken together, these findings underscore the FA pathway's general role in facilitating replication under normal growth conditions.

The Fanconi Anemia Pathway Protects Replication Forks against Transcription-Induced Fork Collapse

Replication of actively transcribed genes induces local replication stress, and, hence, compromises genome stability (Helmrich et al., 2011; Tuduri et al., 2009). We wondered whether the defects we observed in FANCD2-deficient cells could stem from transcription impeding replisome progression. Using proximity ligation assays (PLAs), we observed that FANCD2 colocalizes with total as well as elongating RNA polymerase II (Figure 2A). This colocalization is decreased upon transcription inhibition, with the majority of the PLA-positive cells being confined to the S-phase of the cell cycle (Figures S1B and S1C). Next, we addressed whether transcription contributes to the FA pathway activation and genome instability seen in FANCD2-depleted cells by inhibiting transcription with cordycepin, a potent inhibitor of RNA chain elongation (Rose et al., 1977). Cordycepin treatment significantly decreased FA pathway activation, as measured by the frequency of FANCD2 focus-positive cells as well as FANCD2 monoubiquitination (Figure 2B; Figure S1D). The dose of cordycepin used extensively inhibited transcription without appreciably altering the cell cycle profile (Figures S3E–S3G). Importantly, treatment with two additional transcription inhibitors, 5,6-dichloro-1-β-D-ribofuranosyl-benzimidazole (DRB) and flavopiridol, also greatly decreased the number of FANCD2 focus-positive cells (Figure 2C). Strikingly, inhibition of transcription restored sister fork symmetry in FANCD2-depleted cells to the level observed in the control (Figures 2D and 2E), and this correlated with a reduction in the DNA damage response (Figures 2F and 2G).

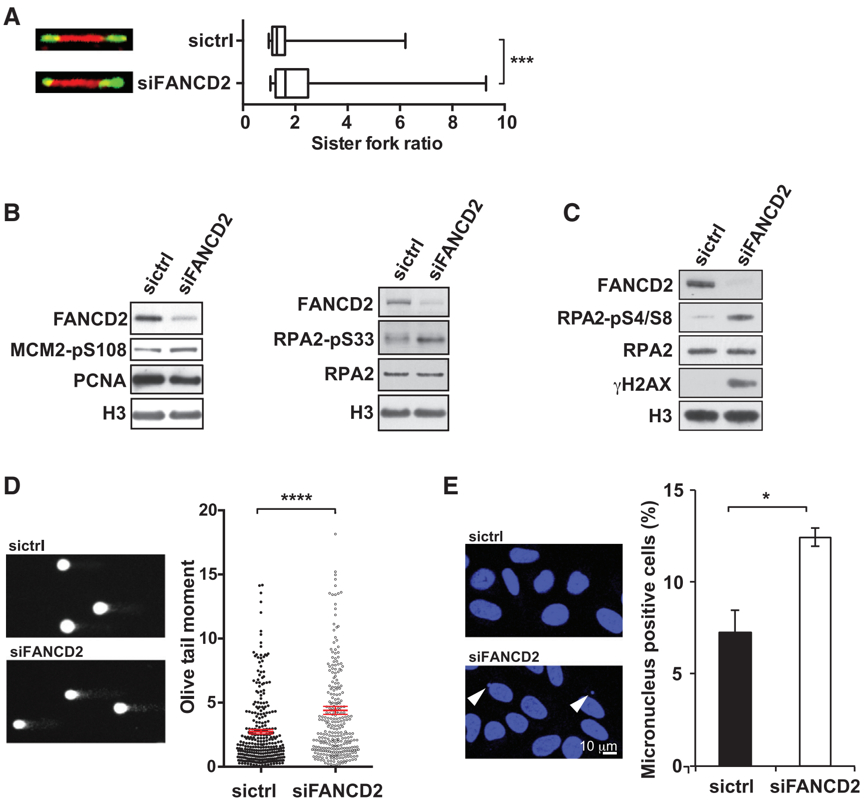

Figure 1. FANCD2 Protects Cells from Replication Stress and Preserves Genomic Integrity

(A) DNA fiber analysis comparing sister fork symmetry. Shown are typical sister forks of U2OS cells treated with control (sictrl) or FANCD2 siRNA (siFANCD2). The ratios of the lengths of two corresponding sister replication forks are plotted. The middle line represents the median and the boxes the 25th and 75th percentiles. The whiskers mark the smallest and largest values. Mann-Whitney test was used to determine statistical significance (n = 3). ***$p \leq 0.001$.

(B) Western blots of whole-cell lysates of control and FANCD2 siRNA-treated U2OS cells probed for phosphorylation of MCM2 on Ser-108 (MCM2-pS108) and RPA2 on Ser-33 (RPA2-pS33). Histone H3, PCNA, and RPA2 served as loading controls.

(C) Western blot showing activation of the DDR upon depletion of FANCD2, including phosphorylation of histone H2AX on Ser-139 (γH2AX) and of RPA2 on Ser-4 and Ser-8 (RPA2-pS4/S8). RPA2 and histone H3 were used as loading controls.

(D) Comet assays of RNAi-treated U2OS cells. Individual data points of olive tail moment are plotted, showing mean ±SEM in red (n = 3, two-tailed Mann-Whitney test). ****$p \leq 0.0001$.

(E) DAPI-stained nuclei and micronuclei (arrowheads) of RNAi-treated U2OS cells. Mean ±SEM of micronucleus-positive cells are plotted (n = 3; unpaired, two-tailed Student's t test). *$p \leq 0.05$.

Depletion of RNA biogenesis factors such as ASF/SF2 compromises transcription and results in genome instability (Li and Manley, 2005; Luna et al., 2005). Therefore, we hypothesized that ASF/SF2 knockdown should increase the likelihood of replication forks colliding with stalled transcription complexes and, as such, further compromise genome stability in FANCD2-depleted cells. Accordingly, we found that ASF/FANCD2 double-depleted cells grew significantly slower (Figure 3A; Figure S2A) and showed increased genome instability, as indicated by an elevated frequency of micronuclei and chromosomal aberrations compared with any of the single depletions (Figures 3B and 3C). These results provide further support to the notion that the defective DNA replication and genome instability observed in FANCD2 knockdown cells are associated with transcription complexes acting as promiscuous replication fork barriers.

The FA Pathway Suppresses Genomic Instability Associated with Unscheduled Accumulation of DNA:RNA Hybrids

Perturbation of transcription is associated with excessive R-loop formation and has recently been linked to genome instability (Aguilera and García-Muse, 2012; Gan et al., 2011; Skourti-Stathaki and Proudfoot, 2014; Tuduri et al., 2009). To determine whether the FA pathway is required to limit such structures, we blotted genomic DNA from control and FANCD2-depleted cells onto a membrane and probed it with the S9.6 antibody, which specifically recognizes DNA:RNA hybrids (Boguslawski et al., 1986). The intensity of the DNA:RNA hybrid signal was increased in FANCD2-depleted cells (Figure 4A). We confirmed this finding by performing immunostaining experiments with the S9.6 antibody and also noticed a marked increase in the nuclear fluorescence intensity in cells depleted of FANCD2 (Figure 4B). We also found increased nuclear DNA:RNA levels when excluding nucleolar signals from the analysis (Figure S2B), indicating that RNA:DNA hybrids are elevated both in the nucleoli and the nucleus.

To understand whether suppression of DNA:RNA hybrids is a general function of the FA pathway, we analyzed additional FA mutants. To this end, we depleted FANCA, which is required for FANCD2 monoubiquitination, and we found significantly increased DNA:RNA hybrid formation in these cells (Figure 4C; Figures S2C and S2D). To verify and extend this observation, we also analyzed the level of DNA:RNA hybrids in avian *FANCD2*$^{-/-}$ and *FANCL*$^{-/-}$ DT40 mutants. The levels of DNA:RNA hybrids were increased in these mutants compared with wild-type cells (Figure 4D), suggesting that accumulation of DNA:RNA hybrids is a general phenomenon associated with FA deficiency. The presence of increased levels of DNA:RNA hybrids in *FANCL*$^{-/-}$ knockout cells suggests that monoubiquitination of FANCD2 is required to suppress their formation because FANCL is the E3 ligase that carries out FANCD2 activation by monoubiquitination (Alpi et al., 2008; Meetei et al., 2003). Therefore, it was not surprising that avian FANCD2 knockin cells expressing endogenous levels of the monoubiquitination-defective FANCD2 K563R mutant protein (Seki et al., 2007) showed higher

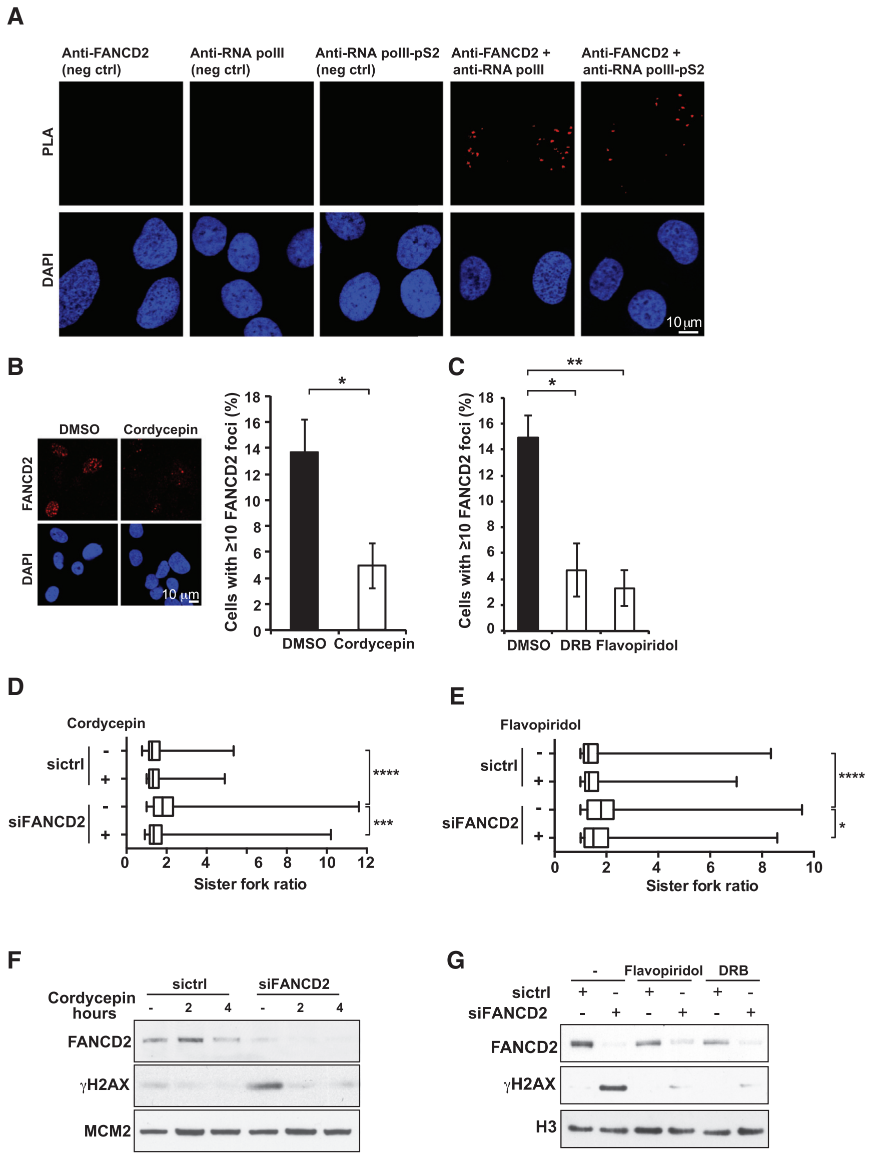

Figure 2. FANCD2 Colocalizes with Sites of Transcription and Prevents Transcription-Induced Replication Stress

(A) Proximity ligation assay showing that FANCD2 colocalizes with total and transcriptionally elongating (phospho-S2) RNA polymerase II. neg. ctrl., negative control.

(B) Treatment with 50 μM of the transcription inhibitor cordycepin for 3 hr decreases the number of FANCD2 focus-positive cells. Means ±SEM are displayed (n = 4; unpaired, two-tailed Student's t test). *p ≤ 0.05.

(C) Treatment with either 100 μM DRB or 0.8 μM flavopiridol for 2 hr decreases the number of FANCD2 focus-positive cells. Means ±SEM are shown (n = 3; unpaired, two-tailed Student's t test). *p ≤ 0.05, **p ≤ 0.01.

(D) DNA fiber analysis comparing sister fork symmetry in control or FANCD2-depleted U2OS cells treated with DMSO or cordycepin. The ratios of the lengths of two corresponding sister replication forks are plotted. The middle line represents the median and the boxes the 25th and 75th percentiles. The whiskers mark the smallest and largest values. Mann-Whitney test was used to determine statistical significance (n = 3). ***p ≤ 0.001, ****p ≤ 0.0001.

(E) Flavopiridol treatment reduces replication fork asymmetry. The experiments were plotted and statistical analysis was performed as in(D). *p ≤ 0.05, ****p ≤ 0.0001.

(F) Western blot of whole-cell lysates from cordycepin- and siRNA-treated U2OS cells. Cordycepin abolishes the activation of γH2AX occurring in the absence of FANCD2. MCM2 was the loading control.

(G) Western blot of whole-cell lysates from control and FANCD2-depleted cells treated with the transcription inhibitor DRB or flavopiridol. H3 served as the loading control.

levels of DNA:RNA hybrids compared with the level observed in wild-type cells (Figure 4D). Taken together, our findings indicate that FA pathway activation and FANCD2 monoubiquitination are required to limit DNA:RNA hybrid formation.

Next, we tested whether DNA:RNA hybrids contribute to the genome instability associated with FA deficiency. To this end, we made use of RNase H1, a nuclease that specifically removes such hybrids. First, we verified that overexpression of RNase H1 reduces the elevated DNA:RNA hybrid load observed in FANCD2 knockdown cells (Figure 5A). We confirmed this observation by incubating genomic DNA purified from control and FANCD2 knockdown cells with RNase H1. As expected, this treatment abolished the DNA:RNA hybrid signal in both samples (Figure S3A). Importantly, RNase H1 overexpression attenuated activation of the FA pathway in cells undergoing normal cell cycle progression, as judged by the significantly diminished formation of FANCD2 focus-positive cells as well as FANCD2 monoubiquitination (Figure 5B; Figure S3B), while not considerably altering the cell cycle profile (Figure S3C). Furthermore, it also rescued impaired the replication fork progression seen in FANCD2 knockdown cells (Figure 5C). This was accompanied by a significant reduction in DNA breaks as well as diminished activation of the DDR response (Figures 5D and 5E). Replicative stress has recently been linked to progressive elimination of hematopoietic stem and progenitor cells in FA patients because of constitutive activation of the p53/p21 response (Ceccaldi et al., 2012). We found that FANCD2-depleted U2OS cells also show a similar response, which was decreased upon overexpression of RNase H1 (Figure 5E). This indicates that physiologically occurring DNA:RNA hybrids induce FA pathway activation and, in its absence, contribute to the constitutive activation of the p53/p21 axis as well as genome instability associated with this disease.

Recently, it has been shown that DNA:RNA hybrid-associated DSB formation is dependent on XPF (Sollier et al., 2014). Therefore, we knocked down XPF in FANCD2-deficient cells to test the contribution of this structure-specific nuclease to the DNA

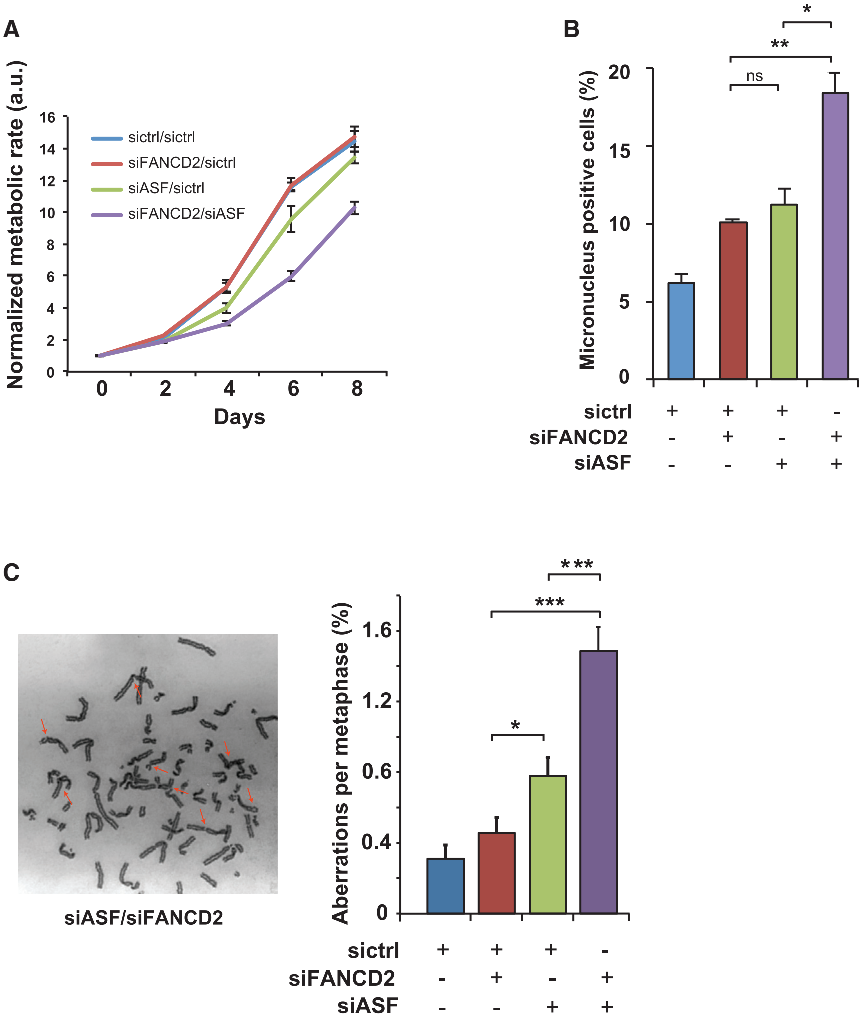

Figure 3. Transcription-Associated Stress Exacerbates the Phenotype of FANCD2-Depleted Cells

(A) Representative alamar blue assay showing that mutual downregulation of ASF and FANCD2 decreases cell proliferation compared with control or single knockdown cells. Error bars show SD from triplicates. a.u., arbitrary unit.

(B) Quantification of micronuclei in U2OS cells showing mean ±SEM of three independent assays. Student's t test was used for statistical analysis. $^{*}p \leq 0.05$; $^{**}p \leq 0.01$; ns, not significant.

(C) Typical example of a metaphase spread of cells treated with siRNA against ASF and FANCD2. Arrows point to aberrations. The graph displays the frequency of chromosomal aberrations in U2OS cells after treatment with the indicated siRNAs (SEM of three independent experiments; Student's t test was used for statistical analysis). $^{*}p \leq 0.05$, $^{***}p \leq 0.001$.

damage load observed in these cells. As shown previously, knockdown of XPF decreased the overall level of DNA breaks, as measured by comet assay (Sollier et al., 2014; Figures S3D and S3E). Concomitant depletion of both XPF and FANCD2 resulted in a slightly decreased level of DNA breaks compared with the siFANCD2 sample. However, the level of damage seen in the double knockdown was still significantly higher than in XPF-depleted cells (Figure S3D). Therefore, we conclude that FANCD2 is required to suppress DNA breaks associated with the presence of DNA:RNA hybrids in a manner that is partially independent of the role of XPF in this process, perhaps specifically during the S-phase of the cell cycle. Finally, to verify our small interfering RNA (siRNA) data, we used clustered regularly interspaced short palindromic repeats (CRISPR)/Cas9 nickase-based gene editing (Hsu et al., 2014) in U2OS cells to generate *FANCD2*$^{-/-}$ clones (Figures S4A and S4B). The use of Cas9 nickase has been shown recently to minimize any off-target effects (Shen et al., 2014). As expected, deletion of FANCD2 in the analyzed clones rendered the cells hypersensitive to the crosslinking agent cisplatin (Figure S4C). Similar to what we observed in siRNA-treated U2OS cells, both *FANCD2*$^{-/-}$ clones displayed increased DNA breaks, genome instability, and DNA:RNA hybrid formation under normal growth conditions (Figures S4D–S4F).

Given that DNA-damaging agents, such as camptothecin (CPT) and UV light, induce DNA:RNA hybrid formation (Sollier et al., 2014; Tresini et al., 2015), we analyzed whether aldehydes, which have recently been implicated in the pathology of FA (Langevin et al., 2011), could also promote the formation of such structures. First, we assessed the effect of low, non-toxic doses of formaldehyde on transcription and cell cycle progression. Treatment with 5 μM formaldehyde for 2 hr did not markedly reduce overall transcription efficiency and cell cycle progression (Figures S5A and S5B). However, we found that formaldehyde treatment resulted in a further increase in DNA:RNA hybrids in FANCD2-deficient cells (Figure 5F). In line with the putative role for formaldehyde in DNA:RNA induction in FANCD2-deficient cells, inhibition of transcription with flavopiridol decreased the level of DNA:RNA hybrids in these cells (Figure S5C). Therefore, formaldehyde toxicity in FA-deficient cells could be, at least partially, related to its ability to induce DNA:RNA hybrids, thereby impacting replisome stability in the absence of FA. Taken together, these data suggest that the FA pathway prevents the deleterious effects associated with DNA:RNA hybrid accumulation and that such structures could be the cause of genome instability in FA-defective cells.

The Fanconi Anemia Pathway Promotes Genome Stability through FANCM-Coupled Resolution of DNA:RNA Hybrids

Next, we asked whether the FA pathway could provide enzymatic activity to resolve DNA:RNA hybrids directly. A likely member of the FA pathway with such a putative function is FANCM. It

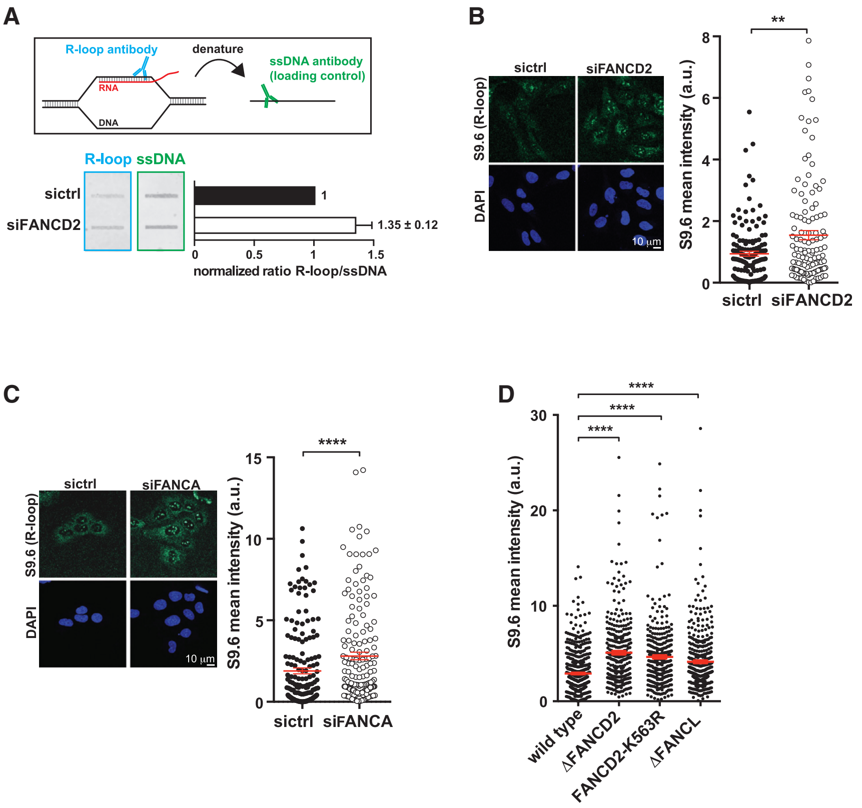

Figure 4. The FA Pathway Prevents the Accumulation of DNA:RNA Hybrids

(A) Experimental overview. Quantitative infrared (IR) fluorescence of genomic DNA of control and FANCD2-depleted cells using S9.6 antibody. Denatured DNA was probed with an antibody against single-stranded DNA (ssDNA) to determine DNA loading. The graph shows the ratio of DNA:RNA hybrid IR fluorescence intensity divided by single-stranded DNA IR fluorescence intensity from four independent experiments.

(B) Assembled z stacks of immunofluorescence staining with S9.6 antibody of FANCD2-depleted or control U2OS cells. Shown is the distribution of mean fluorescence intensity of individual nuclei in arbitrary units, with mean ±SEM in shown red (two-tailed Mann-Whitney test, n = 3). **p $\leq$ 0.01.

(C) The same as in (B) but with cells incubated with sictrl or siFANCA. ****p $\leq$ 0.0001.

(D) Quantified immunofluorescence intensity with the DNA:RNA hybrid-specific antibody S9.6 of wild-type (WT) DT40, *FANCD2*$^{-/-}$, FANCD2 K563R mutant, and *FANCL*$^{-/-}$ cells. The dot plot represents the mean fluorescence intensity of individual nuclei from three independent experiments, with the middle line representing the mean and whiskers the SEM (two-tailed Mann-Whitney test). ****p $\leq$ 0.0001.

possesses double-stranded DNA translocase activity implicated in the processing of Holliday junction intermediates and replication fork reversal in vitro (Gari et al., 2008). In vivo, the protein has been shown to rescue stalled forks (Blackford et al., 2012; Schwab et al., 2010). Studies using recombinant FANCM have tested its activity only with DNA:DNA substrates (Gari et al., 2008). However, the protein is, in fact, classified to belong to the DEAD/DEAH family of DNA:RNA helicases. Therefore, we considered the possibility that FANCM could directly remove DNA:RNA hybrids through its translocase activity. In line with this notion, we observed a significant increase in DNA:RNA hybrid formation in FANCM-depleted cells (Figure 6A; Figure S6A). Importantly, purified FANCM was not only able to unwind replication fork structures, as shown previously (Gari et al., 2008; Figure S6B), but it efficiently unwound DNA:RNA hybrids in vitro (Figures 6B and 6C) despite such substrates being more stable than DNA:DNA hybrids found at a replication fork (Chien and Davidson, 1978). The branch-migratable structures were designed to mimic both the 5′ and 3′ ends of a DNA:RNA hybrid, and our biochemical analyses have shown that FANCM can translocate along either the Watson or Crick strand in a 3′-5′ direction and disrupt DNA:RNA base pairing (Figures 6B and 6C). As expected, the resolution of DNA:RNA hybrids requires FANCM's translocase activity because the translocase-dead mutant protein was unable to unwind these substrates. Similarly, addition of non-hydrolysable ATP (ATP-γ-S) blocked the reaction (Figures 6B and 6C; Figure S6B). Finally, knockin DT40 cells expressing the translocase-dead variant of FANCM (Rosado et al., 2009) also displayed elevated levels of DNA:RNA hybrids (Figure 6D). This suggests a mechanism by which FANCM directly promotes DNA:RNA hybrid resolution, replication fork restart, and, consequently, faithful genome duplication. Because we observed no unwinding activity when the RNA sequence and flap sequence were heterologous (Figure S6C), we conclude that DNA:RNA hybrid resolution is carried out via its branch migration activity.

DISCUSSION

Although DNA:RNA hybrids form naturally and have an important role in various biological processes, such as class-switch recombination or transcription termination (Skourti-Stathaki et al., 2011; Yu et al., 2003), it has recently become apparent that their persistent presence can drive genomic instability (Aguilera and García-Muse, 2012; Skourti-Stathaki and Proudfoot, 2014). Consequently, their formation and removal must be controlled and balanced carefully to prevent a detrimental effect on genome stability, cell survival, and organismal development. Our data suggest that the FA pathway is an important player in controlling DNA:RNA hybrid-associated defects. Accordingly, we show that, under normal growth conditions and in the absence of a functional FA pathway, cells display signs of replicative stress because of replication forks being stalled by transcription complexes, which subsequently leads to replisome collapse, DNA breaks and genome instability. These phenotypes are suppressed by inhibition of transcription or removal

CellPress

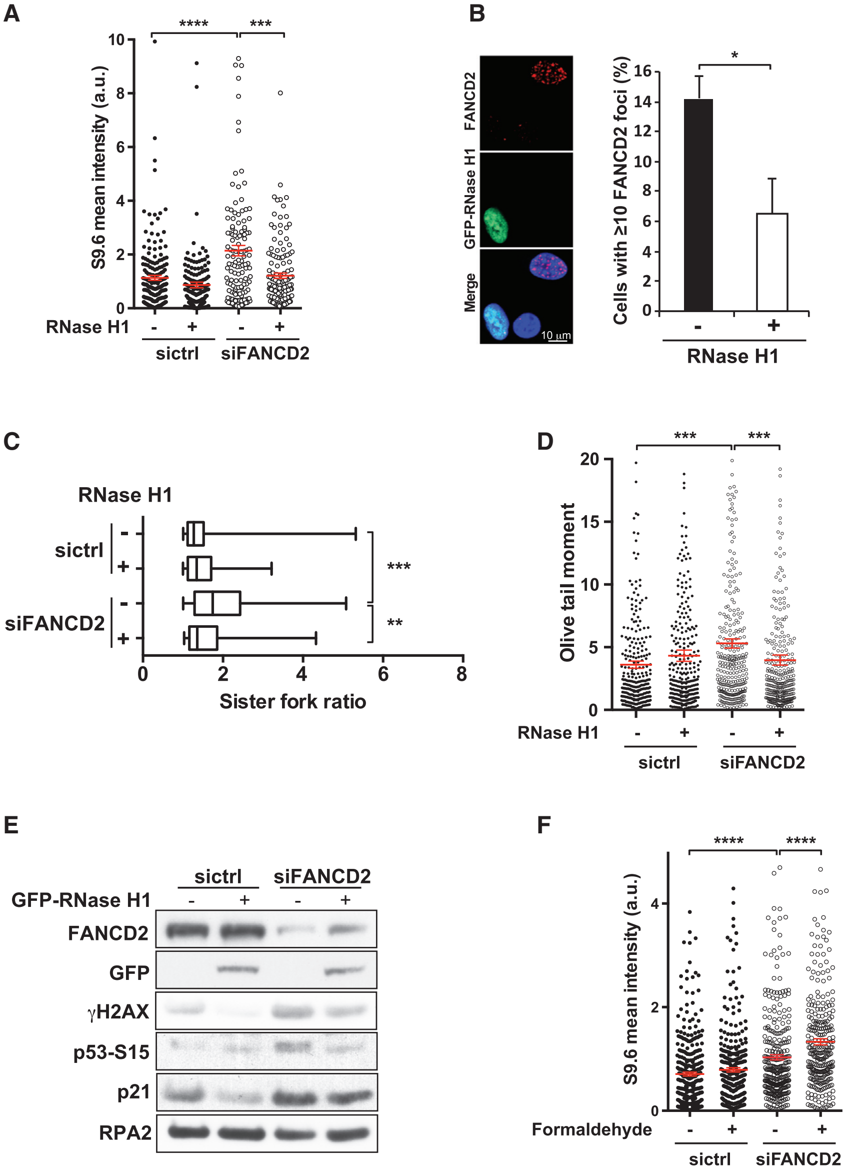

Figure 5. Removal of DNA:RNA Hybrids Prevents Transcription-Induced Replication Stress in FANCD2-Depleted Cells

(A) Mean nuclear DNA:RNA hybrid fluorescence intensity of RNaseH1-overexpressing sictrl or siFANCD2-treated cells (n = 3, mean ±SEM, two-tailed Mann-Whitney test). ***p ≤ 0.001, ****p ≤ 0.0001.

(B) Frequency of cells with more than ten FANCD2 foci in control or GFP-RNaseH1-transfected U2OS cells. Mean ±SEM are plotted (two-tailed Student's t test, n = 5). *p ≤ 0.05.

(C) Sister fork ratio with or without RNaseH1 overexpression. Box and whiskers are as in Figure 1A (n = 3, two-tailed Mann-Whitney test). **p ≤ 0.01, ***p ≤ 0.001.

(D) Comet assay with sictrl and siFANCD2-treated cells overexpressing RNaseH1. Mean ±SEM is shown in red (two-tailed Mann-Whitney test, n = 3). ***p ≤ 0.001.

(E) Western blot showing decreased activation of γH2AX, p53 (S-15) and p21 in FANCD2-depleted cells overexpressing RNaseH1. RPA2 served as a loading control.

(F) Distribution of mean fluorescence intensity of individual nuclei from control and FANCD2-depleted cells in the presence or absence of formaldehyde, with mean ±SEM shown in red (two-tailed Mann-Whitney test, n = 3). ****p ≤ 0.0001.

of DNA:RNA hybrids through overexpression of RNase H1. Mechanistically, the FA pathway not only suppresses the formation of R-loops but also actively resolves such structures utilizing FANCM's translocase activity. Based on these observations, we propose that the FA pathway plays a dual role in suppressing genome instability associated with conflicts between replication and transcription machineries. On one hand, it contributes to the stabilization of stalled replication forks until DNA:RNA hybrids are removed, and, on the other hand, it provides enzymatic activity to directly dismantle them. Consequently, this allows arrested replisomes to restart and faithfully complete genome duplication (Figure 6E).

Mutations in proteins controlling DNA:RNA hybrid levels have been identified in various tumors and are also highly prevalent in leukemias (Groh and Gromak, 2014), occurring at a high rate in FA patients (Kee and D'Andrea, 2012). Therefore, it is conceivable that the FA pathway suppresses tumorigenesis by promoting the resolution of transcription-dependent replication blockades that could otherwise initiate the collapse of replication forks. This hypothesis could also explain the characteristic stem cell defects and heightened risk of tumorigenesis of FA patients because increased fork collapse might affect specific cellular compartments harboring cells that are particularly sensitive to DNA damage, such as hematopoietic precursors. In support of this, constitutive activation of the p53/p21 axis because of physiologically occurring replicative stress has recently been proposed as a central mechanism for progressive elimination of hematopoietic stem cells in FA patients (Ceccaldi et al., 2012). Accordingly, FANCA-deficient mouse hematopoietic stem cells show high levels of DNA damage during progression through S-phase (Walter et al., 2015).

Recently, seminal work from the Patel laboratory has suggested naturally derived aldehydes as drivers of bone marrow failure in FA-deficient mice (Langevin et al., 2011). Because aldehydes generate a plethora of DNA adducts, it is still impossible to precisely pinpoint the nature of the endogenous DNA lesion induced by aldehydes upon which the FA proteins act. Interestingly, our data show that treatment with formaldehyde results in increased DNA:RNA hybrid formation, suggesting a mechanism by which these compounds could contribute to genome instability, in particular in the absence of FA. Notably, treatment

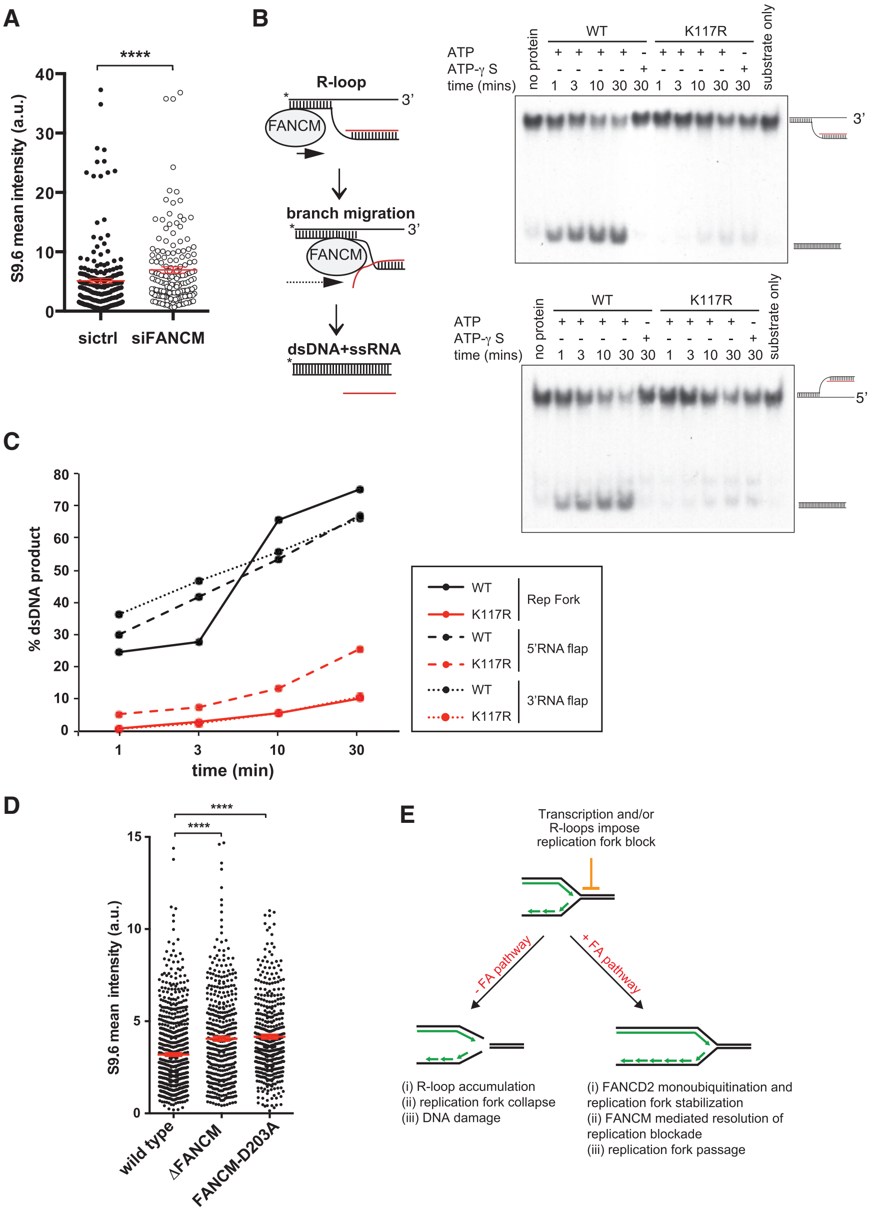

Figure 6. The FA Pathway Prevents DNA:RNA Hybrid Accumulation via the DNA:RNA Branch Migration Activity of FANCM

(A) Quantified immunofluorescence intensity with the DNA:RNA hybrid-specific antibody S9.6 of sictrl or siFANCM-treated cells. The dot plot represents the mean fluorescence intensity of individual nuclei from three experiments, with the middle line representing the mean and whiskers the SEM (two-tailed Mann-Whitney test). ****$p \leq 0.0001$.

(B) Model of branch migration activity of FANCM, leading to the resolution of DNA:RNA hybrids, and in vitro unwinding assays with purified FANCM or FANCM K117R mutant and FAAP24 on migratable 3′ and 5′ DNA:RNA flap structures.

(C) Quantification of DNA:RNA hybrid resolution using a migratable replication (Rep) fork substrate as a positive control.

(D) Quantified immunofluorescence intensity with the DNA:RNA hybrid-specific antibody S9.6 of WT DT40, *FANCM*$^{-/-}$ cells, and DT40 cells expressing FANCM D203A translocase-dead mutant protein. The dot plot represents mean fluorescence intensity of individual nuclei from three independent experiments, with the middle line representing the mean and whiskers the SEM (two-tailed Mann-Whitney test). ****$p \leq 0.0001$.

(E) Model explaining how the FA pathway prevents conflicts between replication and transcription. In the absence of the FA pathway, conflicts between replication and transcription result in activation of the DDR, DNA:RNA hybrid accumulation, defects in replication fork progression, DNA lesions, and genomic instability. In the presence of a functional FA pathway, transcription-induced replication fork stalling leads to monoubiquitination of FANCD2 by the FA core complex proteins and, therefore, activation of the FA pathway, resulting in stabilization of stalled replication forks. Subsequently, FANCM resolves replication blocks consisting of DNA:RNA hybrids via its translocase activity, and replication can resume normally.

with other DNA-damaging agents, such as CPT or UV light, has recently been shown to also induce R-loop formation (Sollier et al., 2014; Tresini et al., 2015). Therefore, toxicity associated with aldehydes could, at least partially, arise from altered transcription and/or transcript splicing, which can induce DNA:RNA hybrid formation (Huertas and Aguilera, 2003; González-Aguilera et al., 2008; Tresini et al., 2015). Alternatively, DNA nicks generated during the repair of aldehyde adducts could drive the formation of such hybrids. Indeed, it has been reported recently that nicks in the DNA template serve as strong DNA:RNA hybrid-initiating sites (Roy et al., 2010). Furthermore, it is possible that multiple compounds that arise endogenously from cellular metabolism could directly or indirectly induce DNA:RNA hybrid formation. Accordingly, DNA damage arises spontaneously in FA-deficient cells, including hematopoietic stem cells (Walter et al., 2015), which are still proficient for aldehyde-detoxifying enzymes. Therefore, we propose that the FA pathway counteracts physiologically arising replicative stress associated with transcription complexes and/or unresolved DNA:RNA hybrids acting as potent replication barriers.

EXPERIMENTAL PROCEDURES

Cell Culture and Transfection with siRNA and DNA

U2OS and HeLa cells were maintained in DMEM and 10% fetal bovine serum (FBS). Chicken DT40 cells were cultured as described before (Schwab et al., 2010). siRNAs from Invitrogen were used for all knockdowns, with the following sequences: FANCA, AAGGGUCAAGAGGGAAAAAUA (Sato et al., 2012); FANCD2, GGUCAGAGCUGUAUUAUUC (Wagner and Karnitz, 2009); FANCM, AGACAUCGCUGAAUUUAAA (Xue et al., 2008); siASF, GUAUUGACCU UAUACUAAA (Tuduri et al., 2009); sictrl, CGUACGCGGAAUACUUCGA

(Tuschl, 2006); and siXPF, UUAACGUGGUGCAUCAAGG. Cells were transfected twice with 24 nM siRNA oligonucleotides using HiPerFect (QIAGEN). Cells were harvested 48 hr after the second siRNA administration. For experiments with RNase H1, transfections were performed 24 hr after the second siRNA pulse, and cells were harvested 36 hr later. 0.8 ng/μl GFP-RNase H1 plasmid DNA was used for experiments analyzing FANCD2 ubiquitylation status, and 0.3 ng/μl was transfected for all other experiments. Transfections were performed with Lipofectamine 2000 (Invitrogen). The GFP-RNase H1 plasmid was a gift from N. Proudfoot.

Chromosomal Aberrations

Cells were prepared for analysis of chromosomal aberrations as described previously (Blackford et al., 2015).

Generation of FANCD2 by CRISPR/Cas9

The following guide RNA (gRNA) sequences targeting the fourth exon of FANCD2 were selected using the optimized CRISPR Design tool (http://crispr.mit.edu; Hsu et al., 2013; gRNA1, TTTGTCTTGTGAGCGTCTGC; gRNA2, GGAGTCTTACATTGAGGATG). DNA oligonucleotides were purchased from Integrated DNA Technologies and cloned into the pX335-GFP vector (Cong et al., 2013) to generate targeting constructs that were subsequently co-transfected in an equimolar ratio into U2OS cells using Lipofectamine 2000. 24 hr after transfection, cells were sorted using a MoFlo cell sorter (Beckman Coulter) for cells expressing Cas9 nickase (GFP-positive cells) and left to recover for 6 days before sorting for single cells and allowing colonies to form. FANCD2 expression was analyzed by western blotting. Two clones showing loss of all detectable FANCD2 were selected for subsequent analysis.

Immunofluorescence Microscopy

U2OS cells were grown overnight on coverslips. DT40 cells were allowed to set on Polysine slides (Thermo Scientific) for 10 min before fixation. Blocking was performed with 10% FBS in PBS for 1 hr. All antibodies were diluted in 0.1% FBS in PBS, and washes were performed with PBS unless stated otherwise. Alexa Fluor 488 or Alexa Fluor 555 secondary antibodies (Molecular Probes) were diluted 1:500. For quantification of micronuclei, cells were fixed and permeabilized in 100% methanol at −20°C for 20 min, blocked, and then incubated with anti-α-tubulin (1:1,000). The coverslips were mounted onto a microscope slide with Vectashield containing DAPI (Vector Laboratories). To visualize GFP-RNase H1 and FANCD2 foci, cells were fixed with 4% paraformaldehyde for 10 min and permeabilized with 0.5% Triton X-100 for 5 min. After blocking, cells were incubated with anti-FANCD2 (1:750) or anti-γH2AX (1:750), followed by incubation with the secondary antibody. Finally, coverslips were incubated with GFP booster (1:200, Atto488, Chromotek) and then mounted. For quantification of the S9.6 mean fluorescence intensity, cells were fixed with 4% paraformaldehyde for 10 min, extracted with 100% methanol at −20°C for 5 min, and then blocked with 5% BSA and 0.2% milk in PBS for 1 hr. S9.6 (1:60) was incubated in blocking buffer for 3 hr, and washes were performed with PBS containing 0.1% Tween 20. Images were acquired with a Zeiss 510 Meta laser-scanning confocal microscope at ×63 magnification. ImageJ was used for picture processing, assembly of z stacks, and quantification of S9.6 mean intensity.

DNA Slot Blot Analysis

3–5 × 10^6 cells were washed in PBS and lysed overnight in DNA lysis buffer (100 mM Tris-HCl [pH 8.5], 5 mM EDTA, 0.2% SDS, and 100 mM NaCl) containing 0.5 mg/ml proteinase K at 55°C. Genomic DNA was precipitated with isopropanol, spooled onto a rod, washed with 70% ethanol, air-dried, and resuspended in Tris-EDTA (TE) buffer. Equal amounts of DNA were blotted onto a nitrocellulose membrane (Amersham Biosciences) using a slot blot apparatus (Bio-Rad) and subsequently baked at 80°C for 2 hr. The membrane was blocked with 5% skimmed milk in PBS and incubated with S9.6 antibody, followed by incubation with an infrared dye secondary antibody (LI-COR Biosciences). The membrane was scanned using a quantitative fluorescence imaging system (Odyssey, LI-COR Biosciences). Subsequently, the membrane was incubated in denaturing buffer (0.4 M NaOH and 0.6 M NaCl), followed by neutralizing buffer (1.5 M NaCl and 0.5 M Tris [pH 7.4]) and an anti-single-strand DNA antibody to detect total DNA.

DNA Fiber Assay

The assay was performed as described elsewhere in detail (Schwab and Niedzwiedz, 2011).

Antibodies, Western Blotting, Cell Cycle Analysis, Transcription Inhibition, and PLA

See Supplemental Experimental Procedures for details.

Biochemical Analysis

Purification of FANCM:FAAP24 was performed as described previously (Coulthard et al., 2013). DNA:RNA hybrids that mimic the 5′ or 3′ region of the transcription bubble were generated using 30-base pair (bp) and 60-bp DNA and RNA oligonucleotides (Table S1). 5 pmol 5′-[γ32P]-labeled XOmig1 and 15 pmol cold oligos were annealed in 50 μl annealing buffer (5 mM Tris-HCl [pH 7.5], 10 mM NaCl, 1 mM MgCl2, and 0.1 mM DTT) using a two-step assembly method according to Table S2 and purified as described previously (Rass and West, 2006). For branch migration assays, 15-μl reactions contained 0.5 nM protein and 0.25 nM DNA substrate in reaction buffer (6 mM Tris [pH 7.5], 5% glycerol, 0.1 mM EDTA, 1 mM DTT, and 0.5 mM MgCl2) and 1 mM ATP or ATP-γ-S. Reactions were carried out at 30°C for the indicated periods, deproteinized, and separated by 12% PAGE in Tris-borate-EDTA (TBE). Quantification was performed using ImageJ after subtracting the background level of double-stranded DNA (dsDNA) in the input. See Tables S1 and S2 for oligo sequences.

Statistics

Statistical analysis was performed using GraphPad Prism 6.0e software and the tests described in the figure legends.

SUPPLEMENTAL INFORMATION

Supplemental Information includes Supplemental Experimental Procedures, six figures, and two tables and can be found with this article online at http://dx.doi.org/10.1016/j.molcel.2015.09.012.

AUTHOR CONTRIBUTIONS

R.A.S. carried out the majority of experimental work with contributions from J.N., W.N., J.L., D.L.M., C.C.L., and M.A.C. F.S. and A.J.D. performed the biochemical analysis. R.J.G. contributed to the DNA:RNA hybrid experiments. W.N. and R.A.S. conceived the project and wrote and edited the manuscript.

ACKNOWLEDGMENTS

We thank Profs. K.J. Patel and M. Takata and Dr. G. Stewart for cell lines and Profs. A. Harris, K.J. Patel, P. McHugh, and N.J. Proudfoot for plasmids, antibodies, and siRNA. We thank Dr. D. Waithe (Wolfson Imaging Centre, Oxford) for his help with analysis of fluorescence immunostainings. We also thank Profs. K.J. Patel and J. Walter for helpful comments on the manuscript. This work was funded by a Worldwide Cancer Research International Fellowship and a WIMM/Medical Research Council Senior Non-Clinical Fellowship (to W.N.), Royal Society Grant UF100717 (to M.A.C.), a Goodger scholarship (to C.C.L.), and MRC and Clarendon scholarships (to D.L.M.). R.J.G's research is supported by the Medical Research Council (grant H4R00121-H40D) A.J.D. is a National Breast Cancer Foundation fellow supported by funding from NHMRC, the Fanconi Anemia Research Fund, and the Victorian Government IOS program.

Received: April 22, 2015
Revised: June 20, 2015
Accepted: September 16, 2015
Published: October 22, 2015

REFERENCES

Aguilera, A., and García-Muse, T. (2012). R loops: from transcription byproducts to threats to genome stability. Mol. Cell *46*, 115–124.

Aguilera, A., and Gómez-González, B. (2008). Genome instability: a mechanistic view of its causes and consequences. Nat. Rev. Genet. *9*, 204–217.

Alpi, A.F., Pace, P.E., Babu, M.M., and Patel, K.J. (2008). Mechanistic insight into site-restricted monoubiquitination of FANCD2 by Ube2t, FANCL, and FANCI. Mol. Cell *32*, 767–777.

Andreassen, P.R., D'Andrea, A.D., and Taniguchi, T. (2004). ATR couples FANCD2 monoubiquitination to the DNA-damage response. Genes Dev. *18*, 1958–1963.

Bhatia, V., Barroso, S.I., García-Rubio, M.L., Tumini, E., Herrera-Moyano, E., and Aguilera, A. (2014). BRCA2 prevents R-loop accumulation and associates with TREX-2 mRNA export factor PCID2. Nature *511*, 362–365.

Blackford, A.N., Schwab, R.A., Nieminuszczy, J., Deans, A.J., West, S.C., and Niedzwiedz, W. (2012). The DNA translocase activity of FANCM protects stalled replication forks. Hum. Mol. Genet. *21*, 2005–2016.

Blackford, A.N., Nieminuszczy, J., Schwab, R.A., Galanty, Y., Jackson, S.P., and Niedzwiedz, W. (2015). TopBP1 interacts with BLM to maintain genome stability but is dispensable for preventing BLM degradation. Mol. Cell *57*, 1133–1141.

Boguslawski, S.J., Smith, D.E., Michalak, M.A., Mickelson, K.E., Yehle, C.O., Patterson, W.L., and Carrico, R.J. (1986). Characterization of monoclonal antibody to DNA.RNA and its application to immunodetection of hybrids. J. Immunol. Methods *89*, 123–130.

Ceccaldi, R., Parmar, K., Mouly, E., Delord, M., Kim, J.M., Regairaz, M., Pla, M., Vasquez, N., Zhang, Q.S., Pondarre, C., et al. (2012). Bone marrow failure in Fanconi anemia is triggered by an exacerbated p53/p21 DNA damage response that impairs hematopoietic stem and progenitor cells. Cell Stem Cell *11*, 36–49.

Chien, Y.H., and Davidson, N. (1978). RNA:DNA hybrids are more stable than DNA:DNA duplexes in concentrated perchlorate and trichloroacetate solutions. Nucleic Acids Res. *5*, 1627–1637.

Cong, L., Ran, F.A., Cox, D., Lin, S., Barretto, R., Habib, N., Hsu, P.D., Wu, X., Jiang, W., Marraffini, L.A., and Zhang, F. (2013). Multiplex genome engineering using CRISPR/Cas systems. Science *339*, 819–823.

Constantinou, A. (2012). Rescue of replication failure by Fanconi anaemia proteins. Chromosoma *121*, 21–36.

Conti, C., Saccà, B., Herrick, J., Lalou, C., Pommier, Y., and Bensimon, A. (2007). Replication fork velocities at adjacent replication origins are coordinately modified during DNA replication in human cells. Mol. Biol. Cell *18*, 3059–3067.

Cortez, D., Glick, G., and Elledge, S.J. (2004). Minichromosome maintenance proteins are direct targets of the ATM and ATR checkpoint kinases. Proc. Natl. Acad. Sci. USA *101*, 10078–10083.

Coulthard, R., Deans, A.J., Swuec, P., Bowles, M., Costa, A., West, S.C., and McDonald, N.Q. (2013). Architecture and DNA recognition elements of the Fanconi anemia FANCM-FAAP24 complex. Structure *21*, 1648–1658.

Gan, W., Guan, Z., Liu, J., Gui, T., Shen, K., Manley, J.L., and Li, X. (2011). R-loop-mediated genomic instability is caused by impairment of replication fork progression. Genes Dev. *25*, 2041–2056.

Gari, K., Décaillet, C., Stasiak, A.Z., Stasiak, A., and Constantinou, A. (2008). The Fanconi anemia protein FANCM can promote branch migration of Holliday junctions and replication forks. Mol. Cell *29*, 141–148.

González-Aguilera, C., Tous, C., Gómez-González, B., Huertas, P., Luna, R., and Aguilera, A. (2008). The THP1-SAC3-SUS1-CDC31 complex works in transcription elongation-mRNA export preventing RNA-mediated genome instability. Mol. Biol. Cell *19*, 4310–4318.

Groh, M., and Gromak, N. (2014). Out of balance: R-loops in human disease. PLoS Genet. *10*, e1004630.

Heddle, J.A., Lue, C.B., Saunders, E.F., and Benz, R.D. (1978). Sensitivity to five mutagens in Fanconi's anemia as measured by the micronucleus method. Cancer Res. *38*, 2983–2988.

Helmrich, A., Ballarino, M., and Tora, L. (2011). Collisions between replication and transcription complexes cause common fragile site instability at the longest human genes. Mol. Cell *44*, 966–977.

Hira, A., Yoshida, K., Sato, K., Okuno, Y., Shiraishi, Y., Chiba, K., Tanaka, H., Miyano, S., Shimamoto, A., Tahara, H., et al. (2015). Mutations in the gene encoding the E2 conjugating enzyme UBE2T cause Fanconi anemia. Am. J. Hum. Genet. *96*, 1001–1007.

Hsu, P.D., Scott, D.A., Weinstein, J.A., Ran, F.A., Konermann, S., Agarwala, V., Li, Y., Fine, E.J., Wu, X., Shalem, O., et al. (2013). DNA targeting specificity of RNA-guided Cas9 nucleases. Nat. Biotechnol. *31*, 827–832.

Hsu, P.D., Lander, E.S., and Zhang, F. (2014). Development and applications of CRISPR-Cas9 for genome engineering. Cell *157*, 1262–1278.

Huertas, P., and Aguilera, A. (2003). Cotranscriptionally formed DNA:RNA hybrids mediate transcription elongation impairment and transcription-associated recombination. Mol. Cell *12*, 711–721.

Kee, Y., and D'Andrea, A.D. (2012). Molecular pathogenesis and clinical management of Fanconi anemia. J. Clin. Invest. *122*, 3799–3806.

Knipscheer, P., Räschle, M., Smogorzewska, A., Enoiu, M., Ho, T.V., Schärer, O.D., Elledge, S.J., and Walter, J.C. (2009). The Fanconi anemia pathway promotes replication-dependent DNA interstrand cross-link repair. Science *326*, 1698–1701.

Kottemann, M.C., and Smogorzewska, A. (2013). Fanconi anaemia and the repair of Watson and Crick DNA crosslinks. Nature *493*, 356–363.

Langevin, F., Crossan, G.P., Rosado, I.V., Arends, M.J., and Patel, K.J. (2011). Fancd2 counteracts the toxic effects of naturally produced aldehydes in mice. Nature *475*, 53–58.

Lecona, E., and Fernández-Capetillo, O. (2014). Replication stress and cancer: it takes two to tango. Exp. Cell Res. *329*, 26–34.

Li, X., and Manley, J.L. (2005). Inactivation of the SR protein splicing factor ASF/SF2 results in genomic instability. Cell *122*, 365–378.

Lossaint, G., Larroque, M., Ribeyre, C., Bec, N., Larroque, C., Décaillet, C., Gari, K., and Constantinou, A. (2013). FANCD2 binds MCM proteins and controls replisome function upon activation of s phase checkpoint signaling. Mol. Cell *51*, 678–690.

Luebben, S.W., Kawabata, T., Johnson, C.S., O'Sullivan, M.G., and Shima, N. (2014). A concomitant loss of dormant origins and FANCC exacerbates genome instability by impairing DNA replication fork progression. Nucleic Acids Res. *42*, 5605–5615.

Luna, R., Jimeno, S., Marín, M., Huertas, P., García-Rubio, M., and Aguilera, A. (2005). Interdependence between transcription and mRNP processing and export, and its impact on genetic stability. Mol. Cell *18*, 711–722.

Meetei, A.R., de Winter, J.P., Medhurst, A.L., Wallisch, M., Waisfisz, Q., van de Vrugt, H.J., Oostra, A.B., Yan, Z., Ling, C., Bishop, C.E., et al. (2003). A novel ubiquitin ligase is deficient in Fanconi anemia. Nat. Genet. *35*, 165–170.

Montes de Oca, R., Andreassen, P.R., Margossian, S.P., Gregory, R.C., Taniguchi, T., Wang, X., Houghtaling, S., Grompe, M., and D'Andrea, A.D. (2005). Regulated interaction of the Fanconi anemia protein, FANCD2, with chromatin. Blood *105*, 1003–1009.

Niedzwiedz, W., Mosedale, G., Johnson, M., Ong, C.Y., Pace, P., and Patel, K.J. (2004). The Fanconi anaemia gene FANCC promotes homologous recombination and error-prone DNA repair. Mol. Cell *15*, 607–620.

Oberbeck, N., Langevin, F., King, G., de Wind, N., Crossan, G.P., and Patel, K.J. (2014). Maternal aldehyde elimination during pregnancy preserves the fetal genome. Mol. Cell *55*, 807–817.

Rass, U., and West, S.C. (2006). Synthetic junctions as tools to identify and characterize Holliday junction resolvases. Methods Enzymol. *408*, 485–501.

Rickman, K.A., Lach, F.P., Abhyankar, A., Donovan, F.X., Sanborn, E.M., Kennedy, J.A., Sougnez, C., Gabriel, S.B., Elemento, O., Chandrasekharappa, S.C., et al. (2015). Deficiency of UBE2T, the E2 Ubiquitin Ligase Necessary for FANCD2 and FANCI Ubiquitination, Causes FA-T Subtype of Fanconi Anemia. Cell Rep. *12*, 35–41.

Rosado, I.V., Niedzwiedz, W., Alpi, A.F., and Patel, K.J. (2009). The Walker B motif in avian FANCM is required to limit sister chromatid exchanges but is dispensable for DNA crosslink repair. Nucleic Acids Res. *37*, 4360–4370.

Rose, K.M., Bell, L.E., and Jacob, S.T. (1977). Specific inhibition of chromatin-associated poly(A) synthesis in vitro by cordycepin 5′-triphosphate. Nature *267*, 178–180.

Roy, D., Zhang, Z., Lu, Z., Hsieh, C.L., and Lieber, M.R. (2010). Competition between the RNA transcript and the nontemplate DNA strand during R-loop formation in vitro: a nick can serve as a strong R-loop initiation site. Mol. Cell. Biol. *30*, 146–159.

Sartori, A.A., Lukas, C., Coates, J., Mistrik, M., Fu, S., Bartek, J., Baer, R., Lukas, J., and Jackson, S.P. (2007). Human CtIP promotes DNA end resection. Nature *450*, 509–514.

Sato, K., Ishiai, M., Toda, K., Furukoshi, S., Osakabe, A., Tachiwana, H., Takizawa, Y., Kagawa, W., Kitao, H., Dohmae, N., et al. (2012). Histone chaperone activity of Fanconi anemia proteins, FANCD2 and FANCI, is required for DNA crosslink repair. EMBO J. *31*, 3524–3536.

Schlacher, K., Wu, H., and Jasin, M. (2012). A distinct replication fork protection pathway connects Fanconi anemia tumor suppressors to RAD51-BRCA1/2. Cancer Cell *22*, 106–116.

Schwab, R.A., and Niedzwiedz, W. (2011). Visualization of DNA replication in the vertebrate model system DT40 using the DNA fiber technique. J. Vis. Exp. *56*, e3255.

Schwab, R.A., Blackford, A.N., and Niedzwiedz, W. (2010). ATR activation and replication fork restart are defective in FANCM-deficient cells. EMBO J. *29*, 806–818.

Seki, S., Ohzeki, M., Uchida, A., Hirano, S., Matsushita, N., Kitao, H., Oda, T., Yamashita, T., Kashihara, N., Tsubahara, A., et al. (2007). A requirement of FancL and FancD2 monoubiquitination in DNA repair. Genes Cells *12*, 299–310.

Shen, B., Zhang, W., Zhang, J., Zhou, J., Wang, J., Chen, L., Wang, L., Hodgkins, A., Iyer, V., Huang, X., and Skarnes, W.C. (2014). Efficient genome modification by CRISPR-Cas9 nickase with minimal off-target effects. Nat. Methods *11*, 399–402.

Sirbu, B.M., Couch, F.B., Feigerle, J.T., Bhaskara, S., Hiebert, S.W., and Cortez, D. (2011). Analysis of protein dynamics at active, stalled, and collapsed replication forks. Genes Dev. *25*, 1320–1327.

Skourti-Stathaki, K., and Proudfoot, N.J. (2014). A double-edged sword: R loops as threats to genome integrity and powerful regulators of gene expression. Genes Dev. *28*, 1384–1396.

Skourti-Stathaki, K., Proudfoot, N.J., and Gromak, N. (2011). Human senataxin resolves RNA/DNA hybrids formed at transcriptional pause sites to promote Xrn2-dependent termination. Mol. Cell *42*, 794–805.

Sollier, J., Stork, C.T., García-Rubio, M.L., Paulsen, R.D., Aguilera, A., and Cimprich, K.A. (2014). Transcription-coupled nucleotide excision repair factors promote R-loop-induced genome instability. Mol. Cell *56*, 777–785.

Taniguchi, T., Garcia-Higuera, I., Andreassen, P.R., Gregory, R.C., Grompe, M., and D'Andrea, A.D. (2002). S-phase-specific interaction of the Fanconi anemia protein, FANCD2, with BRCA1 and RAD51. Blood *100*, 2414–2420.

Tresini, M., Warmerdam, D.O., Kolovos, P., Snijder, L., Vrouwe, M.G., Demmers, J.A., van IJcken, W.F., Grosveld, F.G., Medema, R.H., Hoeijmakers, J.H., et al. (2015). The core spliceosome as target and effector of non-canonical ATM signalling. Nature *523*, 53–58.

Tuduri, S., Crabbé, L., Conti, C., Tourrière, H., Holtgreve-Grez, H., Jauch, A., Pantesco, V., De Vos, J., Thomas, A., Theillet, C., et al. (2009). Topoisomerase I suppresses genomic instability by preventing interference between replication and transcription. Nat. Cell Biol. *11*, 1315–1324.

Tuschl, T. (2006). Cotransfection of luciferase reporter plasmids with siRNA duplexes. CSH Protoc. Published online June 1, 2006. http://dx.doi.org/10.1101/pdb.prot4342.

Wagner, J.M., and Karnitz, L.M. (2009). Cisplatin-induced DNA damage activates replication checkpoint signaling components that differentially affect tumor cell survival. Mol. Pharmacol. *76*, 208–214.

Walden, H., and Deans, A.J. (2014). The Fanconi anemia DNA repair pathway: structural and functional insights into a complex disorder. Annu. Rev. Biophys. *43*, 257–278.

Walter, D., Lier, A., Geiselhart, A., Thalheimer, F.B., Huntscha, S., Sobotta, M.C., Moehrle, B., Brocks, D., Bayindir, I., Kaschutnig, P., et al. (2015). Exit from dormancy provokes DNA-damage-induced attrition in haematopoietic stem cells. Nature *520*, 549–552.

Wang, W. (2007). Emergence of a DNA-damage response network consisting of Fanconi anaemia and BRCA proteins. Nat. Rev. Genet. *8*, 735–748.

Xue, Y., Li, Y., Guo, R., Ling, C., and Wang, W. (2008). FANCM of the Fanconi anemia core complex is required for both monoubiquitination and DNA repair. Hum. Mol. Genet. *17*, 1641–1652.

Yu, K., Chedin, F., Hsieh, C.L., Wilson, T.E., and Lieber, M.R. (2003). R-loops at immunoglobulin class switch regions in the chromosomes of stimulated B cells. Nat. Immunol. *4*, 442–451.

Molecular Cell, Volume *60*

Supplemental Information

The Fanconi Anemia Pathway Maintains Genome Stability by Coordinating Replication and Transcription

Rebekka A. Schwab, Jadwiga Nieminuszczy, Fenil Shah, Jamie Langton, David Lopez Martinez, Chih-Chao Liang, Martin A. Cohn, Richard J. Gibbons, Andrew J. Deans, and Wojciech Niedzwiedz

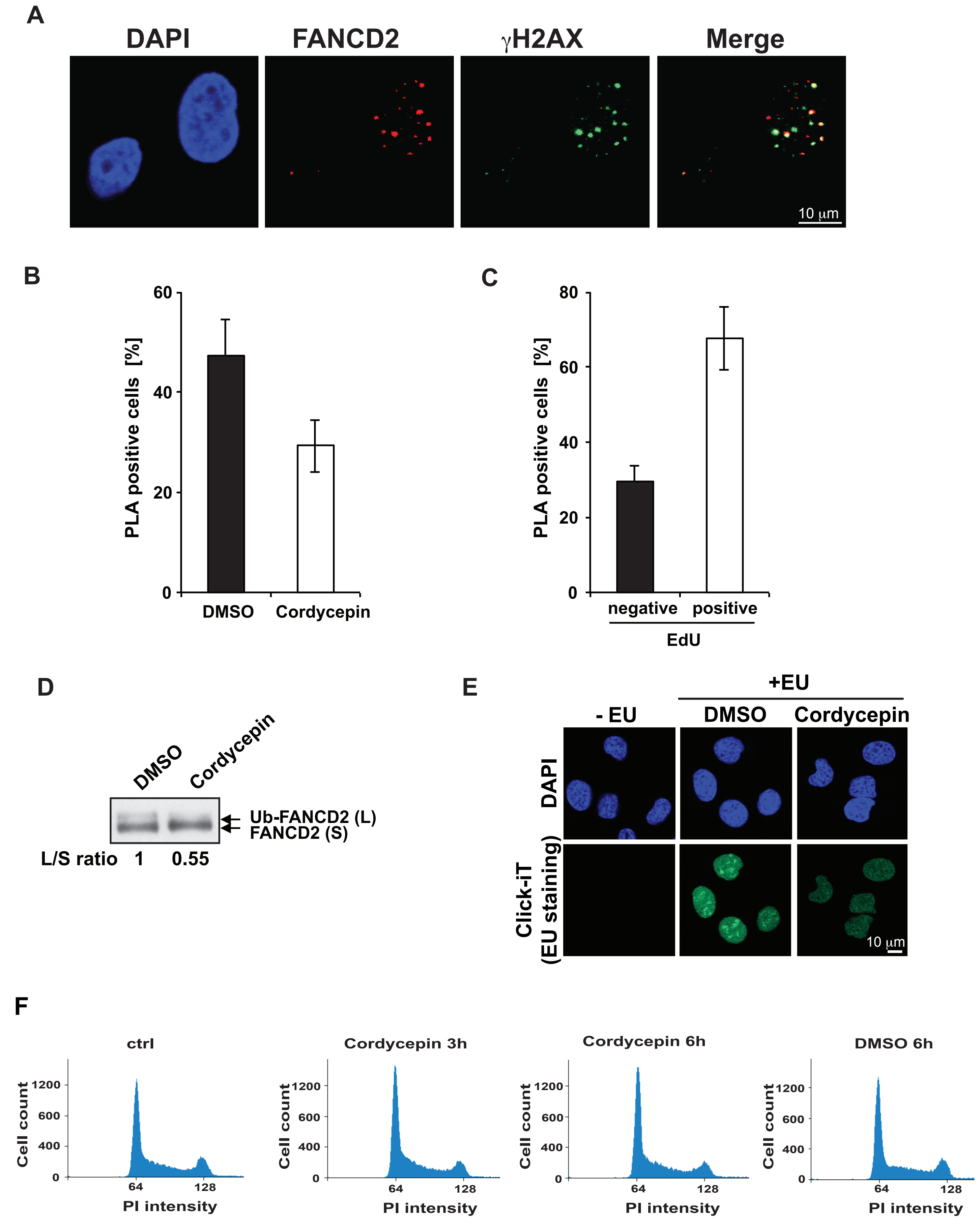

G

	G1	S	G2/M
ctrl	42.7	38.2	17.7
Cordycepin 3h	46.9	38.7	14.2
Cordycepin 6h	44.6	39.2	16.3
DMSO 3h	41.0	38.6	20.3
DMSO 6h	37.8	38.9	23.2

Figure S1

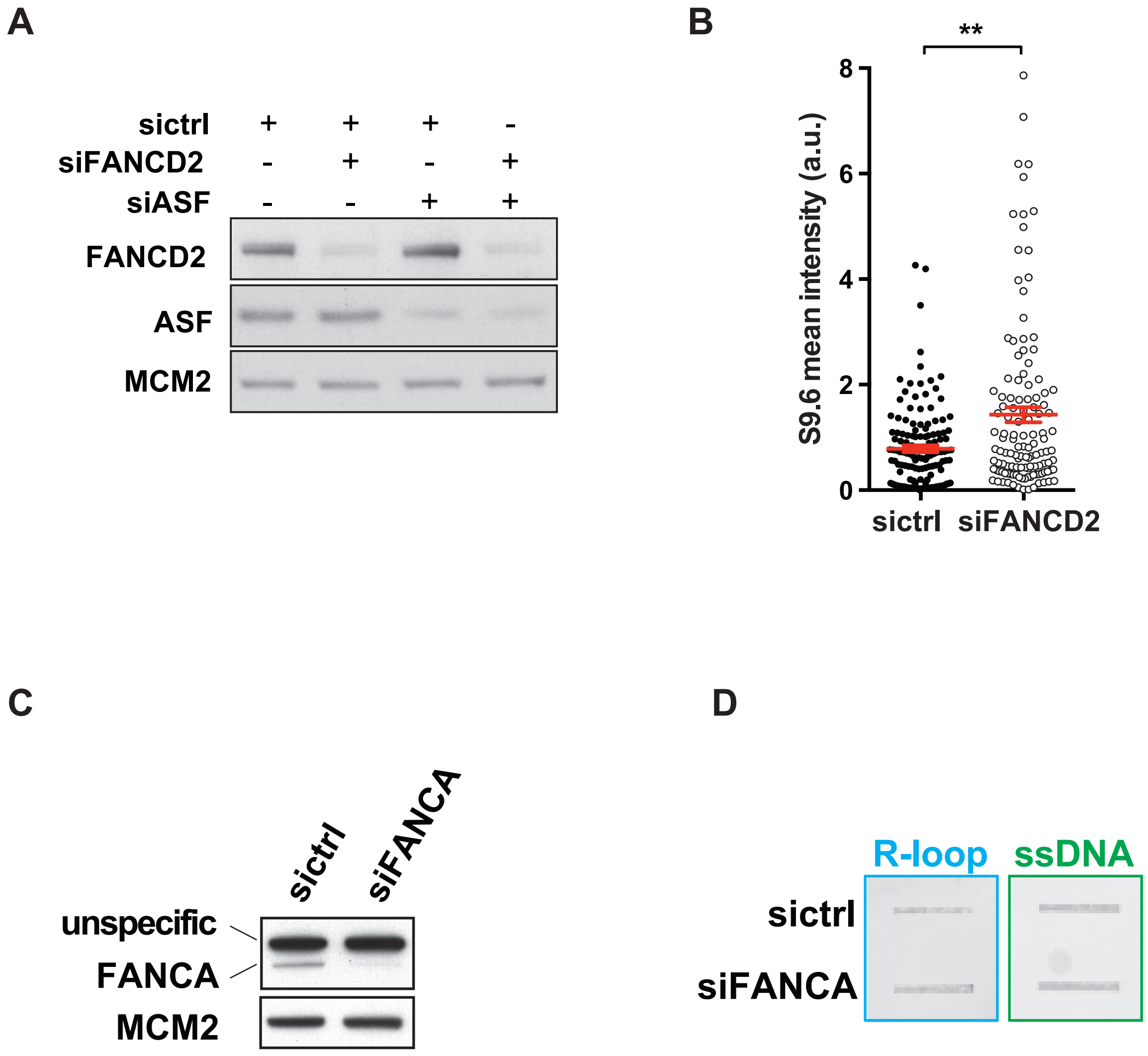
A
sictrl + + + -
siFANCD2 - + - +
siASF - - + +
FANCD2
ASF
MCM2
B
**
S9.6 mean intensity (a.u.)
0
2
4
6
8
sictrl
siFANCD2
C
sictrl
siFANCA
unspecific
FANCA
MCM2
D
R-loop
ssDNA
sictrl
siFANCA

Figure S2

A

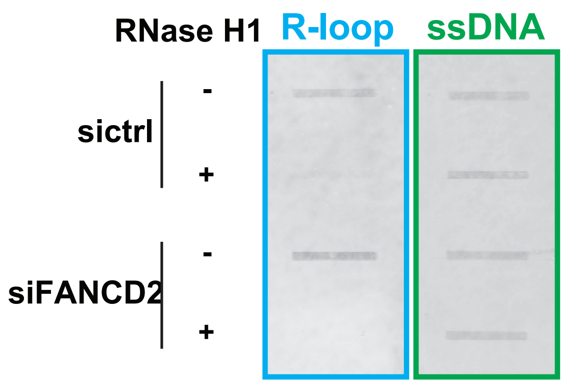

B

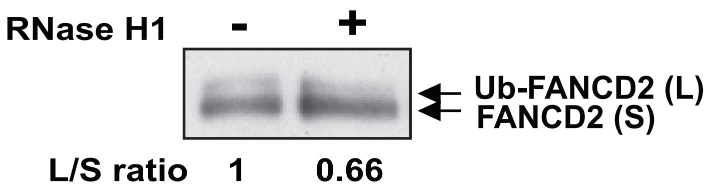

C

	G1	S	G2/M
ctrl	33.15	43.27	24.40
GFP	32.45	43.26	22.03
0.5 μg GFP-RNAse H1	32.16	41.90	25.77
1.5 μg GFP-RNase H1	32.05	43.34	24.49

D

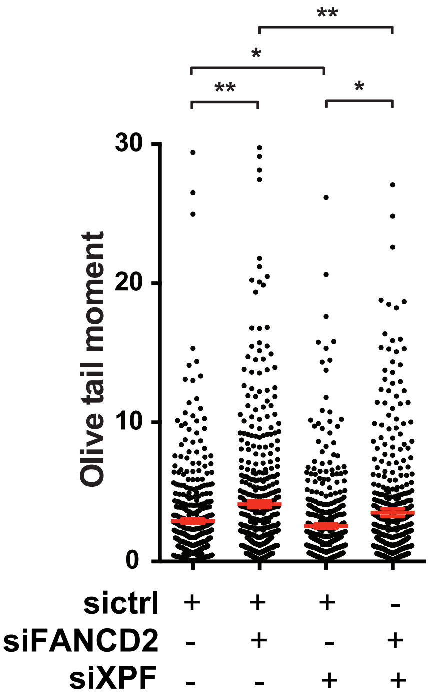

E

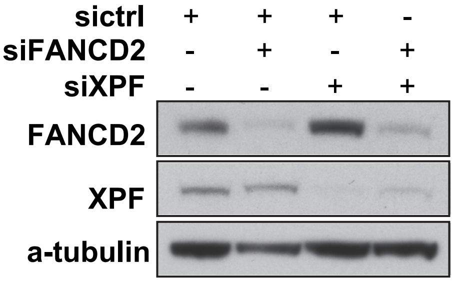

Figure S3

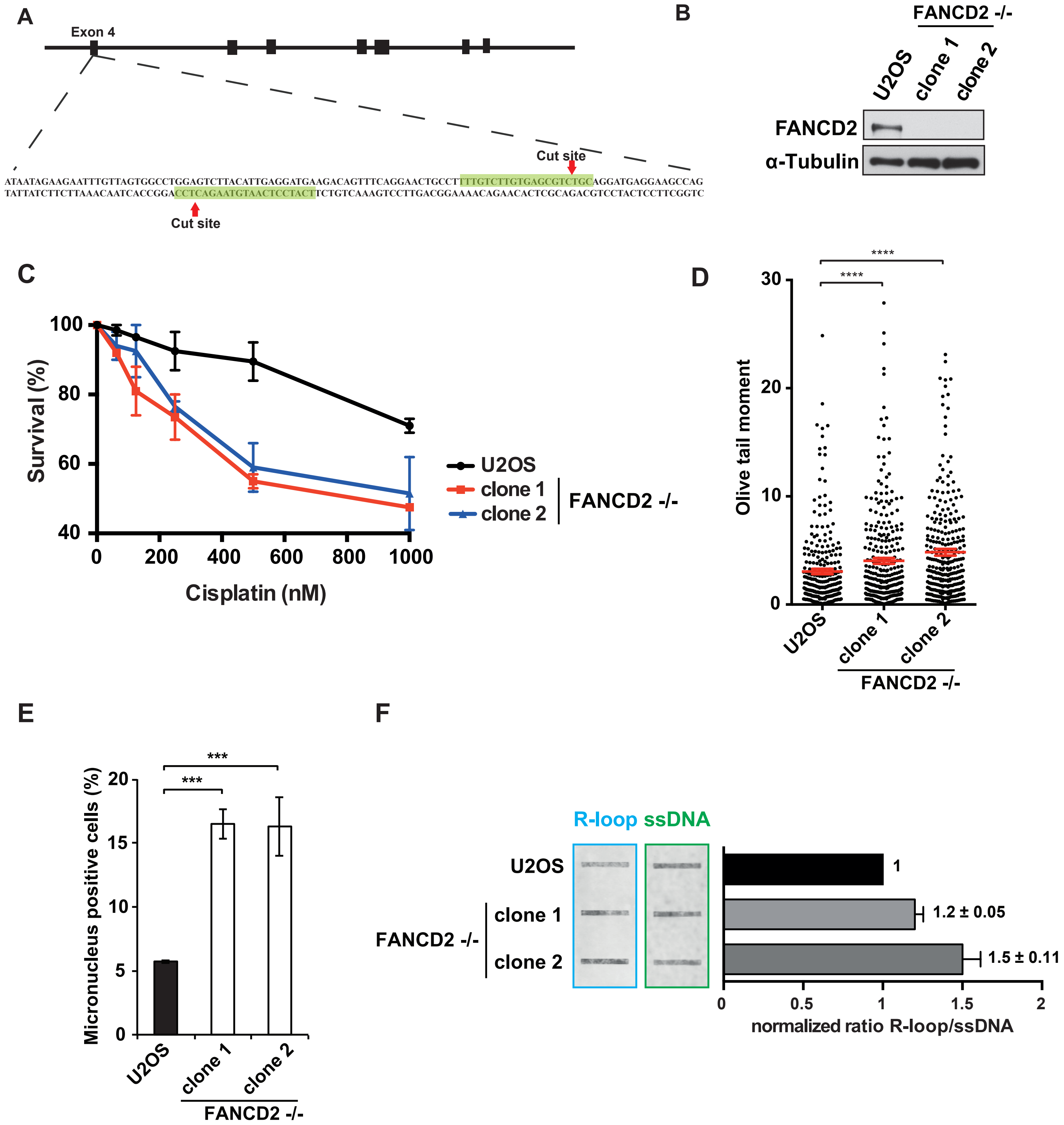
A
Exon 4
ATAATAGAAGAATTTGTTAGTGGCCTGGAGTCTTACATTGAGGATGAAGACAGTTTCAGGAACTGCCTTTTGTCTTGTGAGCGTCTGCAGGATGAGGAAGCCAG
TATTATCTTCTTAAACAATCACCGGACCTCAGAATGTAACTCCTACTTCTGTCAAAGTCCTTGACGGAAAACAGAACACTCGCAGACGTCCTACTCCTTCGGTC
Cut site
Cut site
B
FANCD2 -/-
U2OS
clone 1
clone 2
FANCD2
α-Tubulin
C
Survival (%)
100
80
60
40
0
200
400
600
800
1000
Cisplatin (nM)
U2OS
clone 1
clone 2
FANCD2 -/-
D
Olive tail moment
30
20
10
0
****
****
U2OS
clone 1
clone 2
FANCD2 -/-
E
Micronucleus positive cells (%)
20
15
10
5
0
***
***
U2OS
clone 1
clone 2
FANCD2 -/-
F
R-loop
ssDNA
U2OS
FANCD2 -/-
clone 1
clone 2
1
1.2 ± 0.05
1.5 ± 0.11
0
0.5
1
1.5
2
normalized ratio R-loop/ssDNA

Figure S4

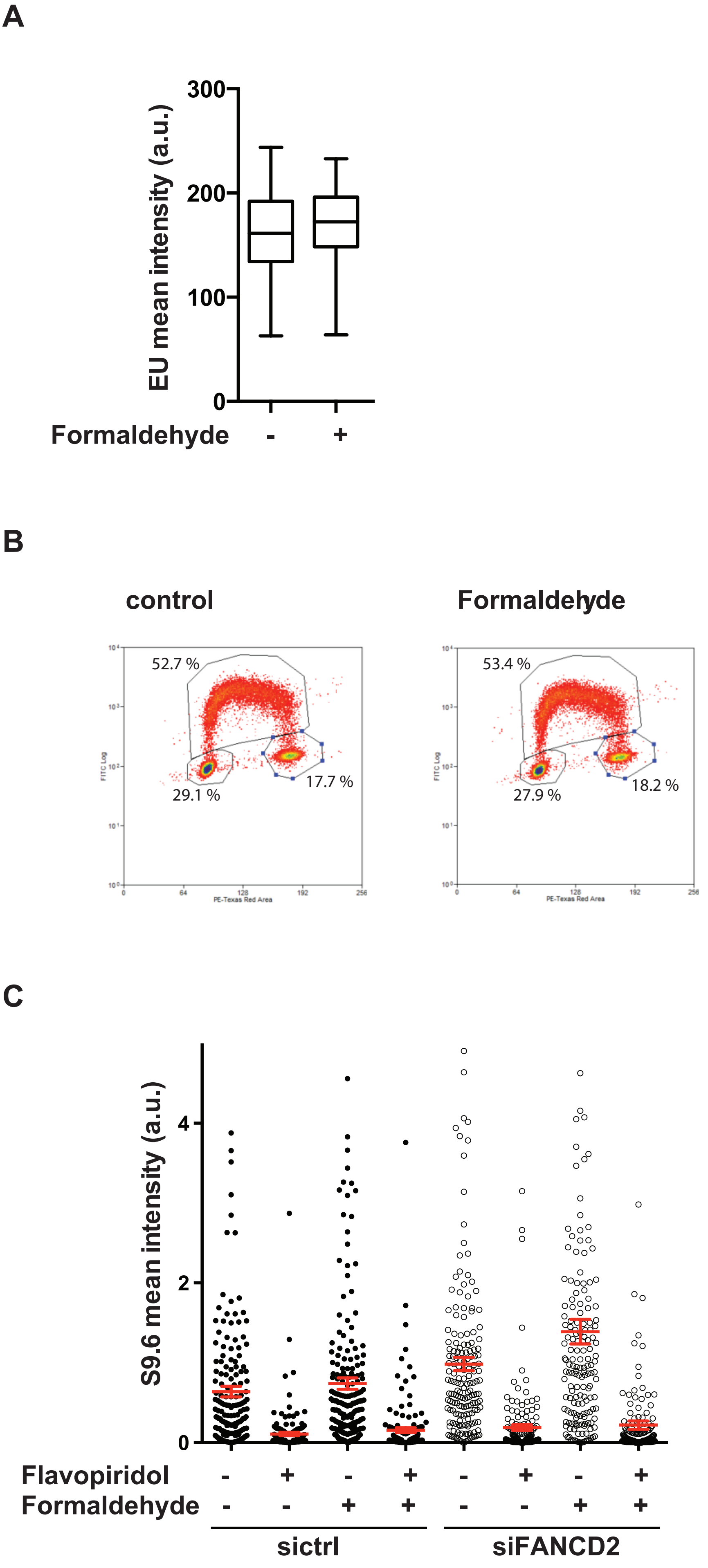
A
EU mean intensity (a.u.)
300
200
100
0
Formaldehyde
-
+
B
control
Formaldehyde
52.7 %
29.1 %
17.7 %
53.4 %
27.9 %
18.2 %
FITC Log
PE-Texas Red Area
C
S9.6 mean intensity (a.u.)
4
2
0
Flavopiridol
Formaldehyde
sictrl
siFANCD2

Figure S5

A

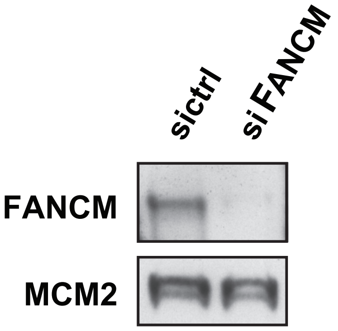

B

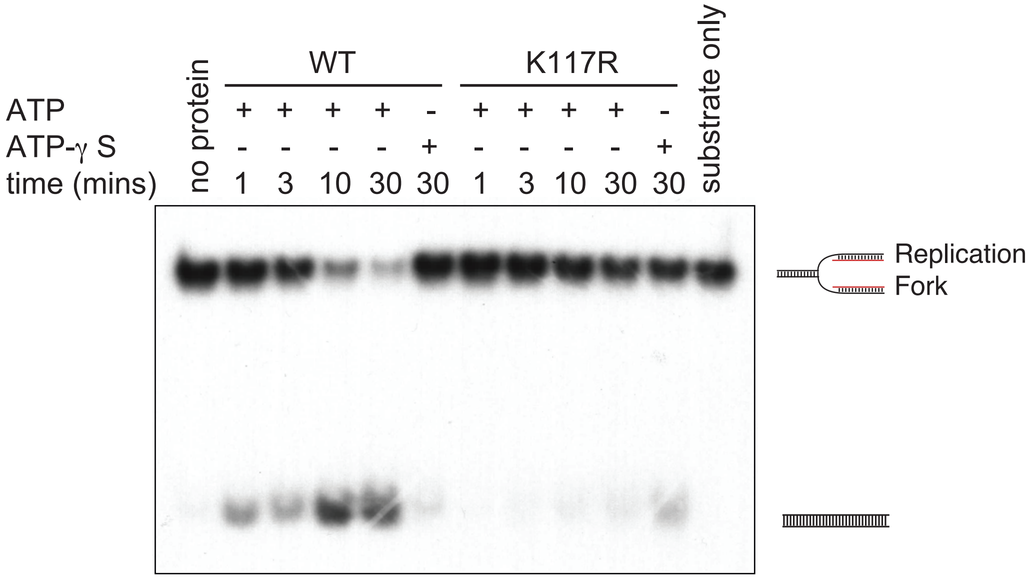

C

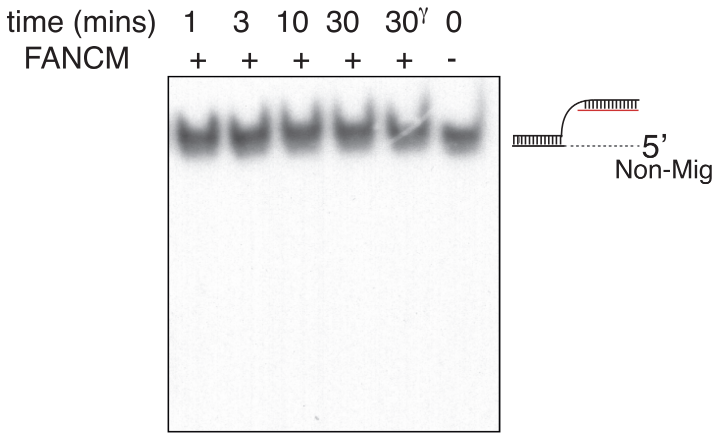

Figure S6

Supplementary Figures:

Figure S1, related to Figure 1 and 2. (A) Colocalization of γH2AX and FANCD2 foci in undamaged cells. Exponentially growing U2OS cells were examined by immunofluorescence using antibodies against FANCD2 and γH2AX. (B) Quantification of PLA assay using antibodies against endogenous FANCD2 and RNA polII-pS2. Graph showing frequency of PLA positive cells in the presence of DMSO or cordycepin (means and SEM, n=3). (C) Chart displaying percentage of PLA positive cells with or without EdU staining (means and SEM, n=3). (D) Effect of cordycepin treatment on FANCD2 ubiquitination. Monoubiquitination of FANCD2 was analyzed in HeLa cells treated with DMSO or 50 μM cordycepin for 2h using an antibody against the full-length protein. Densitometric analysis was used to determine the ratio between the monoubiquitinated (L) and the short form of FANCD2 (S). (E) Efficacy of cordycepin treatment. Analysis of global RNA transcription in cells treated with EU (5-ethynyl uridine) and DMSO or 50 μM cordycepin. Newly synthesized RNA is visualized using the click chemistry and Alexa Fluor 488 azide. (F) Cell cycle profiles of exponentially growing U2OS cells exposed to 50 μM cordycepin for the times indicated analyzed using flow cytometry and propidium iodide staining. (G) Percentage of cells in the indicated phases of the cell cycle.

Figure S2, related to Figure 3 and 4. (A) Western blot of whole cell lysates from U2OS cells treated with sictrl, siFANCD2 or siASF according to the legend. MCM2 serves as loading control. (B) Graph displays quantification of nuclear S9.6 staining intensities excluding nucleolar signals of the same pictures used for the analysis in Figure 4B with mean and SEM in red (two-tailed Mann-Whitney test, n=3). (C) Western blot of whole cell lysates from U2OS cells treated with control or FANCA siRNA. MCM2 is the

loading control. (B) Slot blot of genomic DNA from sictrl and siFANCA treated cells probed with the S9.6 antibody. After denaturation the DNA loading was assessed with an antibody recognizing ssDNA.

Figure S3, related to Figure 4 and 5. (A) Genomic DNA of control and FANCD2-depleted cells was treated with RNase H1 *in vitro* before blotting onto a membrane. Binding of the S9.6 antibody was assessed by IR fluorescence measurement and detection of ssDNA after denaturation serves as loading control. (B) RNase H1 decreases FANCD2 ubiquitination. Monoubiquitination of FANCD2 was analyzed in exponentially growing HeLa cells mock transfected or transfected with a cDNA coding for RNase H1 fused to GFP. An antibody against full-length FANCD2 was used to determine the ratio between monoubiquitinated FANCD2 (L) and the unmodified, short form (S). (C) Percentage of cells in the indicated phases of the cell cycle for control U2OS, U2OS cells transfected with a construct encoding GFP only, and U2OS cells transiently transfected with the indicated concentrations of plasmid DNA encoding GFP-RNase H1. (D) Comet assays of U2OS cells depleted for XPF and FANCD2 by siRNA. Individual data points of olive tail moment are plotted showing mean and SEM in red, n=3, two-tailed Mann-Whitney test. (E) Western blot confirming knock down of FANCD2 and XPF.

Figure S4, related to Figure 1, 2 and 4. (A) FANCD2 knockout generation strategy using the CRISPR-Cas9 nickase. Schematic representation of the human FANCD2 genomic locus with guide RNA sequences highlighted in green and predicted cut sites marked by red arrows. (B) Western blot of whole cell lysates from U2OS cells blotted with an anti-FANCD2 antibody. Tubulin is the loading control. (C) Survival assay of control U2OS and $FANCD2^{-/-}$ clones treated with the indicated doses of cisplatin. Survival data

represent mean +/- SEM, (n=3). (D) Comet assays of WT U2OS and FANCD2$^{-/-}$ clones. Individual olive tail moment values are plotted showing mean and SEM in red, n=3, two-tailed Mann-Whitney test. (E) Means and SEM of micronuclei positive WT U2OS and FANCD2$^{-/-}$ clones are plotted (n=3, unpaired, two-tailed Student's t-test). (F) Slot blot of genomic DNA from sictrl and FANCD2$^{-/-}$ cells probed with the S9.6 antibody. After denaturation the DNA loading was assessed with an antibody recognizing ssDNA.

Figure S5, related to Figure 5. (A) Global RNA transcription was measured in cells treated with EU in the presence and absence of formaldehyde. Fluorescence intensity measurements were derived from newly synthesized RNA visualized using the click chemistry and Alexa Fluor 488 azide. (B) Cell cycle analysis of BrdU labelled cells treated or untreated with formaldehyde. (C) Distribution of mean fluorescence intensity of individual nuclei from control and FANCD2 depleted cell in the presence or absence of formaldehyde and the transcription inhibitor flavopiridol (mean and SEM in red, two-tailed Mann-Whitney test, n=3).

Figure S6, related to Figure 6. (A) Western blot of control and siFANCM treated U2OS cell lysates probed with an antibody against FANCM; MCM2 was used to assess equal protein loading. (B) FANCM and FAAP24 unwind a migratable replication fork structure (control). Unwinding is inhibited in the presence of FANCM K117R helicase-dead mutant protein or non-hydrolysable ATP (ATP-γ-S). (C) FANCM and it's binding partner FAAP24 do not resolve a non-migratable DNA:RNA flap structure.

Supplementary experimental procedures

Antibodies. Anti-MCM2 (ab4461, Abcam), anti-MCM2-pS108 (AHP1525, AbD Serotec), anti-RPA2 (Ab-2; NA18, Calbiochem), anti-ASF (324500, Invitrogen), anti-RPA2-pS4/8 (A300-245A, Bethyl), anti-RPA2-pS33 (A300-246A, Bethyl), anti-PCNA (PC10; sc-56, Santa Cruz), anti-H3 (ab1791, Abcam), anti-γ-H2AX (JBW301; 05-636, Millipore), anti-FANCD2 for western blotting (FI17; sc-20022, Santa Cruz), anti-FANCD2 for IF was a gift from K.J. Patel, anti-FANCA (6512, FARF), anti-FANCM (Cancer Research Technology), anti-GFP (11814460001, Roche), anti-RNA polymerase II (05-623, Millipore), anti-RNA polymerase II H5 (MMS-129R, Covance), anti-single stranded DNA (MAB3034, Millipore), α-tubulin (T5168, Sigma-Aldrich), anti-p53 (phospho-S15, Abcam), anti-p21 (Santa Cruz Biotech), XPF (ab17798, Abcam). The anti-DNA:RNA hybrid (S9.6) antibody was from S9.6 hybridoma cells grown in Iscove's modified Dulbecco's medium with 1x HT solution (HAT minus aminopterine) and 20% horse serum until they reached the stationary phase and began to die. The medium was then filtered and sodium azide added. The S9.6 containing medium was used with no further purification.

Transcription assay. Inhibition of transcription after cordycepin treatment was assessed with the Click-it RNA Imaging Kit and Alexa Fluor 488 azide (Invitrogen). U2OS cells were grown on cover slips and following treatment with 50 μM cordycepin (Sigma-Aldrich) for 2 h, cells were incubated with 1 mM EU for 1 h and then fixed with 4% paraformaldehyde for 10 min. Click reactions were performed in accordance with the manufacturer’s instructions.

Western blot analysis. Whole cell protein extracts were prepared by lysing washed cells in denaturing buffer (9 M urea, 150 mM 2-mercaptoethanol, 50 mM Tris-HCl pH 7.3) and subsequently sonicated to shear genomic DNA. FANCM was separated on 7% Tris-glycine mini gels and FANCD2 ubiquitination was assessed on 3-8% NuPAGE Tris-Acetate mini gels (Life Technologies). For all other proteins 4-12% NuPAGE Bis-Tris mini gels (Life Technologies) were used. Gels were transferred onto nitrocellulose membranes (GE Healthcare). HRP conjugated secondary antibodies (Dako) and Immobilon enhanced chemiluminescence reagents (Millipore) were used to visualize

antigens. Intensities of the bands of the long and short forms of FANCD2 were measured using ImageJ.

Proximity ligation assay (PLA). U2OS cells were grown on cover slips, washed with PBS and fixed with 4% paraformaldehyde for 10 min. After permeabilization with 0.5% Triton X-100 for 5 min, cells were blocked with 10 % FBS for 1 h and then incubated with anti-FANCD2 (1:750) alone as negative control or in combination with either anti-RNA polymerase (1:600) or anti-RNA polymerase II H5 (phospho-S2) (1:600) in 0.1% FBS for 2 h. Binding of PLA probes, ligation and amplification was performed with reagents from the Duolink In Situ Kit (Sigma-Aldrich) according to manufacturer's instructions.

S-phase analysis of PLA positive cells. Cells were labelled with 10 μM EdU for 30 min and then fixed with 10% PFA for 10 min. S-phase cells were visualized using the Click-iT EdU imaging kit from Molecular Probes according to manufacturers instructions. Subsequently, PLA assays were performed as described.

Cell cycle analysis. U2OS cells were treated with DMSO or 50 μM cordycepin for 3 and 6 h, trypsinized and washed with PBS containing 2% FBS. Cells were resuspended in 0.5 ml PBS with 2% FBS and fixed with ice cold 70% ethanol. Cells were then washed with PBS, incubated with 2 M HCl for 30 min, washed twice with PBS containing 0.5% Tween-20 and 1% BSA, and subsequently stained with propidium iodide solution (PBS, 0.5% Tween-20, 1% BSA, 20 mg/ml propidium iodide, 250 mg/ml RNase A) for 30 min at 37°C. Cell cycle profiles were analyzed using CyAn ADP Analyzer (Beckman Coulter) and Summit 4.3 software.

Cell proliferation assay. RNAi treated U2OS cells were plated in 96 well plates and 4 h before fluorometric measurements, alamarBlue (Bio-Rad) was added to the samples according to manufacturer's instructions.

Alkaline comet assay. U2OS cells were trypsinized, washed and resuspended in PBS at a density of 0.5 x 10^6 cells/ml. Cell suspensions were mixed at a 1:1 ratio with 1.5 % low melting point agarose Type VII (Sigma-Aldrich) and cast on a microscope slide precoated with 0.5% agarose Type IA (Sigma-Aldrich). After solidification, slides were

incubated in lysis buffer (2.5 M NaCl, 100 mM EDTA, 10 mM Tris, pH 10, 1% Triton X-100) for 2 h at 4°C. After lysis, slides were placed in a horizontal electrophoresis tank covered by electrophoresis buffer (1 mM EDTA, 300 mM NaOH) and incubated for 40 min. Electrophoresis was performed at 1V/cm distance between electrodes for 40 min. Slides were washed twice with 0.4 M Tris-HCl pH 7.5 and twice with distilled water before immersing in 70% ethanol for 5 min. Slides were then air-dried and subsequently stained with SybrGold (Invitrogen). Comets were analyzed with Komet 6 (Andor) software.

Supplemental Table 1, related to experimental procedures: Oligos used in the generation of DNA:RNA or DNA:DNA hybrid structures. Complementary sequences are colour coded:

XOmig1	5'ACGCTGCCGAATTCTACCAGTGCCTTGCTAGGACATCTTTGCCCACCTGCAGGTTCACCC3'
XOmig 2	5'GGGTGAACCTGCAGGTGGGCAAAGATGTCCCAGCAAGGCACTGGTAGAATTCGGCAGCGT3'
XOFlap3 R	RNA-5'GGACAUCUUUGCCCACCUGCAGGUUCACCC3'
XOFlap5 R	RNA-5'GGGUGAACCUGCAGGUGGGCAAAGAUGUCC3'
XO2non	5'CGATAGTCGGATCCTCTAGACAGCTCCATGTAGCAAGGCACTGGTAGAATTCGGCAGCGT3'

Supplementary Table 2, related to experimental procedures: 2 step annealing procedure for generation of different DNA:DNA and DNA:RNA hybrid structures

Substrate	Step 1		Step 2	
	Oligos	Temp	Oligos	Temp
Migratable Replication fork	(A) XOmig1 + XOFlap5R, (B) XOmig2 + XOFlap3R	100C, O/N	Mix (A) and (B)	37C, 1hr
Migratable 5' Flap	(A) XOmig1 + XOFlap5R,	100C, O/N	Mix (A) and XOmig2	37C, 1hr
Migratable 3' Flap	(B) XOmig2 + XOFlap3R	100C, O/N	Mix (B) and XOmig2	37C, 1hr
dsDNA	(C) XOmig1 + XOmig2	100C, O/N	-	-
Non migratable 5'Flap	(D) XOmig1 + XO1non + XOFlap5R	100C, O/N	-	-
